# Bidirectional flow of MHD nanofluid with Hall current and Cattaneo-Christove heat flux toward the stretching surface

**DOI:** 10.1371/journal.pone.0264208

**Published:** 2022-04-14

**Authors:** Muhammad Ramzan, Zahir Shah, Poom Kumam, Waris Khan, Wiboonsak Watthayu, Wiyada Kumam

**Affiliations:** 1 KMUTT Fixed Point Research Laboratory, Room SCL 802 Fixed Point Laboratory, Science Laboratory Building, Department of Mathematics, Faculty of Science, King Mongkut’s University of Technology Thonburi (KMUTT), Bangkok, Thailand; 2 Center of Excellence in Theoretical and Computational Science (TaCS-CoE), Science Laboratory Building, Faculty of Science, King Mongkut’s University of Technology Thonburi (KMUTT), Bangkok, Thailand; 3 Department of Mathematical Sciences, University of Lakki Marwat, Lakki Marwat, Pakistan; 4 Department of Medical Research, China Medical University Hospital, China Medical University, Taichung, Taiwan; 5 Department of Mathematics and Statistics, Hazara University Mansehra, Khyber Pakhtunkhwa, Pakistan; 6 Applied Mathematics for Science and Engineering Research Unit (AMSERU), Program in Applied Statistics, Department of Mathematics and Computer Science, Faculty of Science and Technology, Rajamangala University of Technology Thanyaburi (RMUTT), Pathum Thani, Thailand; Central University of Karnataka, INDIA

## Abstract

Vacuum pump oil (VPO) is used as a lubricant in pumps of different machines. The rate of heat transport is a fundamental requirement of all phenomena. To enhance the rate of heat transmission and reduce the amount of energy consumed as a result of high temperatures. For this reason, the vacuum pump oil (VPO) is taken as a base fluid and *Fe*_3_*O*_4_ is the nanoparticles suspended in VPO. That’s why, the present study inspected the consequence of Hall current, Joule heating effect and variable thickness on these three-dimensional magnetohydrodynamics bidirectional flow of nanoliquid past on a stretchable sheet. Further, the Cattaneo-Christove heat flux and radiation impacts are also considered. The VPO−*Fe*_3_*O*_4_ nanofluid model is composed of momentum equations in *x*−direction, *y*−direction and temperature equations. The leading higher-order non-linear PDEs of the current study have been changed into non-linear ODEs with the implementation of appropriate similarity transformations. The procedure of the homotopy analysis method is hired on the resulting higher-order non-linear ODEs along with boundary conditions for the analytical solution. The significance of distinct flow parameters on the velocities in *x*−direction, *y*−direction and temperature profiles of the nanofluid have been encountered and briefly explained in a graphical form. Some important findings of the present modelling are that with the increment of nanoparticles volume fraction the nanofluid velocities in *x*−direction and *y*−direction are increased. It is also detected that higher estimations of magnetic field parameter, Prandtl number and thermal relaxation time parameter declined the nanofluid temperature. During this examination of the model, it is found that the *Fe*_3_*O*_4_-Vacuum pump oil (VPO) nanofluid enhanced the rate of heat transfer. Also, the vacuum pump oil (VPO) has many industrial and engineering applications. The current study will help to improve the rate of heat transmission by taking this into account due to which working machines will do better performance and the loss of useful energy will be decayed. Lastly, the skin friction coefficient and Nusselt number are also illustrated in a tabular form. Some major findings according to the numerical computation of the problem are that the enhancing estimations of magnetic parameter, nanoparticles volume fraction and wall thickness parameter augmented the skin friction coefficient in *x*−direction and Nusselt number. The reduction in skin friction coefficient of the nanofluid in *y*−direction is examined for Hall current and shape parameter.

## 1. Introduction

The non-Newtonian fluid is much attention of the scientist and researchers because of its numerous applications over the previous few years in different field of manufacturing and industries. Most important engineering and industrial applications of the non-Newtonian nanofluids are thermal insulations, designing of heat exchangers, food stuff processing, geothermal reservoirs, wire and fiber coating, cooling of nuclear reactor, reactor fluidization and oil recovery etc. As a result, the several scientists and researchers have used non-Newtonian nanofluids in their study. Ahmad et al [1] elaborated the study of Joule heating and magnetic effects over the non-Newtonian thermally radiative Sisko fluid and found that the rising estimations of magnetic parameter amplified the speed of the fluid. Ahmad and Khan [2] employed the bvp4c technique to evaluate the mathematical study of non-Newtonian Sisko nanoliquid flow under the curved stretchable surface. From their conclusion, it can be perceived that the pressure profile of the nanoliquid is elevated for curvature parameter. Ahmad and Khan [3] examined the problem of non-Newtonian Sisko magneto-nanoliquid flow through the appliance of activation energy and heat transport on the moving curved surface. They investigated that the transmission of heat is higher when the parameter of heat source/sink is enhanced. Ahmad et al [4] proposed the mathematical modelling of non-Newtonian Sisko liquid toward the shirking surface along with the effect of Cattaneo-Christov double diffusion. It is obtained that the decrement in ReCf of the liquid is sensed for material parameter. In another study of non-Newtonian liquid, Ali and Sandeep [5] conducted the research on the study of the non-Newtonian Casson ferrofluid along with Cattaneo-Christov heat flux and heat transmission under the rotating cone and wedge. Kumar et al [6] reported the modelling of magnetohydrodynamic flow of non-Newtonian Cattaneo-Christov heat flux with variable heat source/sink over the wedge and cone and discussed that for both wedge and cone the thermal and energy boundary layer thickness are not similar. Saleem et al [7] computed the numerical solution of Cattaneo-Christov heat flux over the three-dimensional non-Newtonian upper-convective Maxwell liquid over the vertical stretching sheet. From this analysis, it is noted that the thermal relaxation time parameter boosted the rate of heat transport.

Nanofluids have been extensively explored in past years because of their vast variety of applications in science and technology. Besides the technology, nanofluid is frequently used in biomedical to target cancer cells via nanoscale drug delivery systems and to identify blood flow blockages in the arteries via thallium scans (radioactive tracer). Nanofluid can also be used to purify waste materials for renewable energy. Furthermore, the nanoliquid offers a vast range of applications in manufacturing and industrial developments including microelectronics devices, hybrid-powered engines, heat exchangers, boiler flue gas temperature reduction, engine cooling thermal management, fuel cells, chiller and domestic refrigerators. Researchers and scientists have conducted a lot of nanofluid investigations, in addition to these various potential applications. Bishnoi et al [8] discoursed the effect of Hall current in a flow of magnetic nanofluid across the two horizontal infinite free boundaries and found that the instability of the system becomes higher with the increasing of Hall current. Qaiser et al [9] explored the Walter-B nanofluid model in the existence of mass transfer and joule heating impacts toward the stretching surface and they employed the bvp4c technique in MATLAB for the numerical outcomes of their problem. Alotaibi et al [10] introduced the existence of the viscous dissipation effect in the numerical investigation of Casson nanofluid through the non-linear extendable sheet. From their results, it is inspected that the liquid Casson parameter reduced the speed of the liquid particles. Upreti et al [11] used the Runge-Kutta scheme for the examination of three-dimensional Darcy-Forchheimer flow of CNTs nanoliquid problem via stretched sheet and observed that the transfer of heat is enhanced when the nanoparticles concentration in the base fluid is increased. Islam et al [12] explored the heat/source-sink behavior in the mixed convention nanofluid through the stretching cylinder. In this work, they examined that the heat source parameter increased the fluid temperature. Pal and Mandal [13] studied the homogeneous-heterogeneous chemical reaction in a water-based carbon nanotubes nanofluid along the stretching surface. They noticed that the skin friction coefficient rises as the porosity and nanoparticle volume fraction increases. Rasool et al [14] deliberated the study of Williamson nanoliquid in a stretchable sheet through the cumulative contribution of activation energy and entropy.

Magnetohydrodynamics played a significant role in solar physics, astrophysics, blood pump machines, vacuum pump laboratory plasma experiments, plasma physics, pumps, bearings, MHD generators and cancer tumors treatments. Additionally, the magnetohydrodynamics has an extensive range of industrial and engineering applications including nuclear reactors, power generators, drugs targeting, plasma stability, molten salts and electromagnetic waves. The researchers employed MHD as a significant tool in their research area based on the aforementioned applications. Jawad et al [15] tested the aspect of heat source and thermal radiation through the converging/diverging channel in a non-Newtonian MHD Casson nanoliquid flow. In this enquiry, they distinguished that the Casson parameter upsurges the liquid velocity. Shah et al [16] debated the magnetohydrodynamics flow of *Ag*−*Cu*/water nanoliquid in the attendance of joule heating behavior, they employed the HAM on the higher-order nonlinear ODEs for the mathematical computation of their model. Tlili et al [17] analyzed the magnetohydrodynamics nanoliquid flow past a thin needle through the occurrence of the Hall effect and entropy generation. Their results indicate that when the Hall current is enhanced the entropy of the liquid is diminished. Hayat et al [18] explicated the occurrence of convective mass and heat conditions on the problem of magnetohydrodynamics flow of third-grade nanoliquid passes through the stretchable surface. Gupta et al [19] described the analytical study of variable thickness and thermal radiation in magnetohydrodynamics flow of Williamson nanoliquid. In this study, they investigated that the radiation parameter escalated the liquid temperature. Sobamowo et al [20] explained the magnetohydrodynamic flow of upper-convective Maxwell viscoelastic nanoliquid toward the channel in a porous media under the slip effects and also, they discussed the role of several parameters over the concentration and temperature profiles of the fluid. Rasool et al [21] disclosed the mathematical modeling of MHD type Casson nanofluid flow in the context of Darcy-Forchheimer relation by using the nonlinear stretchable surface. They perceived that Darcy-Forchheimer reduced the transfer of heat.

From the last few decades, the researchers and scientists are fascinated by the relationship between heat and mass transport in various fluid models because of their different uses in industry and engineering. Its diverse impacts on fluid flow have been explored by various researchers. Biswas and Ahmmed [22] elaborated the performance of heat and mass transmission in a Casson nanoliquid model due to vertical plate along with Hall current and find that skin friction coefficient is raised for enhancing estimations of Brownian motion and Casson parameters. Alreshidi et [23] demonstrates the role of heat and mass transmission in a magnetohydrodynamics three-dimensional nanoliquid flow along with joule heating effect. They make a comparison with the earlier published outcomes and discovered that they were in good agreement. Sreedvei et al [24] employed the most powerful numerical scheme is known as the finite element technique for the investigation of *Au*−*Eg* and *Ag*−*Eg* Maxwell nanoliquid via stretching cylinder under the heat and mass transport impacts. Raghunath et al [25] scrutinized the unsteady magnetohydrodynamics flow in a porous media toward the vertical plates under the heat and mass transmission behavior and their conclusion shows that the Sherwood number is decreased when Schmidt number is enhanced. Yasmin et al [26] surveyed the magnetohydrodynamics flow of the non-Newtonian micropolar fluid problem above the curved stretched sheet including the heat and mass transmission characteristics and found that the fluid concentration is elevated with the increase of the radius of curvature. Gireesha et al [27] inspected the flow of Oldroyd-B nanolquid with heat and mass transport phenomena under the stretchy surface through the utilization of radiation effect. In this work, they obtained that the fluid temperature is higher for greater estimation of radiation parameter. Ullah et al [28] talked about the heat-mass transmission behavior in three-dimensional nanofluid flow in the attendance of activation energy and also, they discussed some physical properties of the nanofluid.

In mechanical, civil and electrical engineering, the variable thick surface has a variety of applications. As a consequence, the variable thickness has attracted the curiosity of scientists and researchers in this period of exploration. Daniel et al [29] evaluated the mathematical modelling of MHD flow of nanoliquid in a stretched surface with variable thickness and joule heating impacts and attained that the concentration boundary layer is a declining function of Lewis parameter. Hayat et al [30] conversed the consequence of variable thickness on the nanoliquid flow through the heated Riga plate. They applied the HAM scheme for the explanation of their mathematical modelling. In another research, Hayat et al [31] made the investigation on the variable thickness of magnetohydrodynamics Powell-Eyring nanofluid past a stretched surface. Furthermore, they explained that the magnetic and power index parameters enhanced the surface drag coefficient. Doh et al [32] questioned the significance of chemical reaction over the flow of nanoliquid along with variable thickness toward the spinning disk. Salahuddin et al [33] focused over the flow of Casson nanoliquid via thin needle with the occurrence of variable thickness. They concluded that, when the Prandtl number is amplified, the augmentation in the Nusselt number is examined. Awais et al [34] offered the Cattaneo-Christov heat flux problem in hydromagnetic mixed convection flow under the wall by using the variable thickness property in which the authors have found the convergence serious solution of the presenting problem.

The Lorentz force defines the force applied on a charged particle traveling through the magnetic field and the Hall impact is simply an extension of that force. An electric potential is created across the conductor that is vertical to both the electric and magnetic fields when a sufficiently strong magnetic field is utilized in the direction vertical to the electric field. It is observed that this whole phenomenon is formed due to the induced magnetic field and is called as the Hall Current. Firstly, the Hall current is described by the American physicist Hall in 1879 [35]. And then Acharya et al [36] applied the Rk-4 method for the demonstration of radiative nanofluid problem past on a rotating disk with Hall current effect in which they depict that the Hall current declined the fluid temperature. Shah et al [37] examined the micropolar nanoliquid model between the two plates with the implementation of the Hall effect. They attained the analytical solution of their model and debated the impacts of different parameters. Ramzan et al [38] dissected the feature of Hall effect over the three-dimensional bioconvective hyperbolic nanoliquid along with activation energy. It is stated that activation energy enhanced the fluid concentration. Ibrahim et al [39] revealed the idea of magnetohydrodynamics flow of Casson nanoliquid with the attendance of Hall effect and slip impacts. They examined that the ReCf is weekend with the variation of slip and Casson parameters. In another study of Hall current, Ibrahim et al [40] utilized the three-dimensional Maxwell nanofluid model for the investigation of Hall current and chemical reaction. In this research work, they computed that the mixed convection parameter enlarges the *Sh*_*x*_. Fiza et al [41] considered the Hall effect behavior over the problem of three-dimensional MHD Jeffery fluid flow in between two parallel plates. Moreover, they found that mass flux is increased at the upper plate and reduced at the lower plate.

From the above-mentioned literature, it is notable that no study is reported on the magnetohydrodynamics bidirectional flow of a nanoliquid with the occurrence of thermal radiation. To fill this gap, the Cattaneo-Christove heat flux and Joule heating effects model in magnetohydrodynamics bidirectional flow of *Fe*_3_*O*_4_-vacuum pump oil (VPO) nanofluid are taken into account. Hall current and variable thickness are also calculated in the designed model. Moreover, the vacuum pump oil (VPO) is used as a base fluid to enhance the rate of heat transmission and *Fe*_3_*O*_4_ is the nanoparticles in VPO. The current problem of nanofluid is molded in form of PDEs and then these PDEs are transmuted into ODEs by using the appropriate similarity variables. In MATHEMATICA 10, the most powerful analytical technique, called homotopy analysis, is exploited on the higher-order ODEs to investigate the influence of distinct flow parameters over the velocities in *x*−direction, *y*−direction and temperature of the nanofluid in a graphical form. Further, the skin friction coefficient and Nusselt number against several flow parameters have been discussed in a tabular form. In the current analysis, the physical significance of the Cattaneo-Christov heat flux is that it is used for the deliberation of heat rate transport phenomena. Also, the energy equation is formulated using the Cattaneo-Christov heat flux model. The significance of thermal relaxation time on the boundary layer can be predicted using the Cattaneo-Christov heat flux model. The Cattaneo-Christov heat flux problem is based on the classical transport model of Fourier law of heat transport. The Current Cattaneo-Christov heat flux problem is useful in different field of applied sciences. It has many applications in fields of applied sciences, industries and biomedical such as electronic devices, hybrid power generators, magnetic drags targeting, nuclear reactor cooling and heat conduction in tissue pasteurization of milk and etc.

## 2. Problem formulation

**Fig 1:**
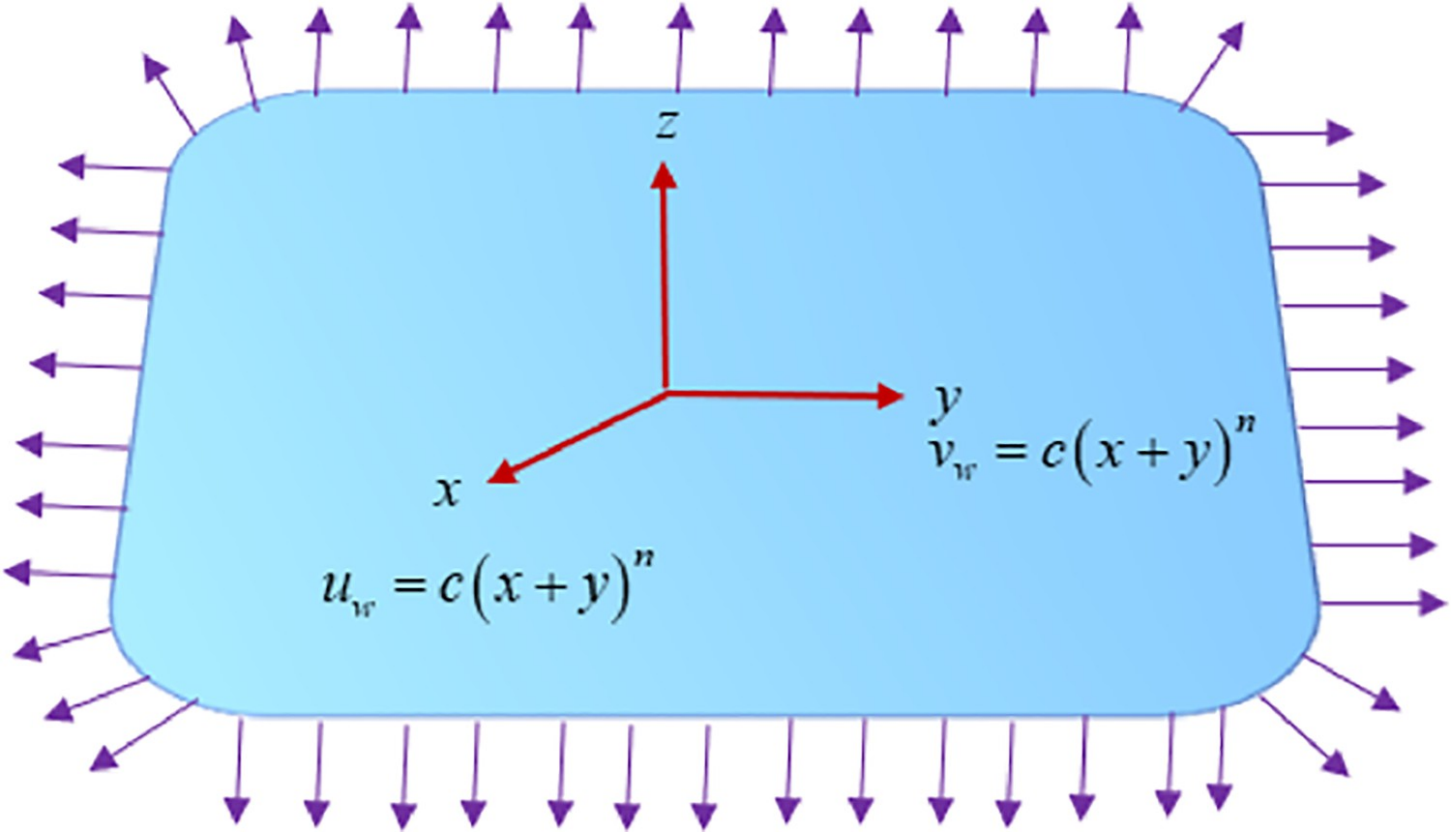
Geometry of the problem.

Let us consider the three-dimensional magnetohydrodynamic bidirectional flow of a nanoliquid with the influence of Joule heating effect toward the stretching surface. The velocities for the stretching sheet are assumed to be *u* = *c*(*x*+*y*)^*n*^ in *x*−direction and *v* = *c*(*x*+*y*)^*n*^ in *y*−direction. The present study consists of *Fe*_3_*O*_4_-vacuum pump oil (VPO) nanoliquid in which vacuum pump oil (VPO) is taken as a base fluid and *Fe*_3_*O*_4_ used as nanoparticles. The uniform magnetic field *B*_0_ has been applied in the current analysis. Variable thickness $z=A{\left(x+y\right)}^{\frac{1-n}{2}}$ for stretching surface is taken into account. Further, the thermal radiation effect and Cattaneo-Christov heat flux are investigated. The surface is kept constant at the uniform temperature *T*_*w*_ and the ambient temperature *T*_∞_. Fig 1 illustrates the problem’s physical significance.

Keeping in mind the above assumptions, the relation between Ohm’s Law and Hall current is given as

$$J+\frac{\omega_{e}\tau_{e}}{B_{0}}\times \left(J\times B\right)=\sigma_{nf}(E+V\times B+\frac{1}{en_{e}}pe),$$ (1)

where the current density vector is denoted by *J* = (*J*_*x*_,*J*_*y*_,*J*_*z*_) in *y*−direction, the magnetic induction is *B*, the electric field intensity is *E*, the velocity vector is designed as *V* = (*u*,*v*,*w*), the effective electrical conductivity is represented by *σ*, *ω*_*e*_ is the electron frequency, e is for electron charge and the electronic pressure is signified by *pe*. The electric field is taken to be zero *E* = 0 because no voltage is executed over the fluid flow. So, the Hall current in components form is taken as

$$J_{x}=\frac{\sigma_{nf}B_{0}^{2}}{\rho_{nf}(1+m^{2})}\left(v-mu\right),$$ (2)

$$J_{y}=\frac{\sigma_{nf}B_{0}^{2}}{\rho_{nf}(1+m^{2})}\left(mu-v\right).$$ (3)

For Hall current *m* = *ω*_*e*_*τ*_*e*_.

The three-dimensional flow equations for the current flow analysis are

$$\frac{\partial u}{\partial x}+\frac{\partial v}{\partial y}+\frac{\partial w}{\partial z}=0,$$ (4)

$$\begin{array}{l} u\frac{\partial u}{\partial x}+v\frac{\partial u}{\partial y}+w\frac{\partial u}{\partial z}=\upsilon_{nf}\frac{\partial^{2}u}{\partial z^{2}}-\frac{\sigma_{nf}B_{0}^{2}}{\rho_{nf}}u+\frac{\sigma_{nf}B_{0}^{2}}{\rho_{nf}(1+m^{2})}(v-mu), \end{array}$$ (5)

$$u\frac{\partial v}{\partial x}+v\frac{\partial v}{\partial y}+w\frac{\partial v}{\partial z}=\upsilon_{nf}\frac{\partial^{2}v}{\partial z^{2}}-[\frac{\sigma_{nf}B_{0}^{2}}{\rho_{nf}}v-\frac{\sigma_{nf}B_{0}^{2}}{\rho_{nf}(1+m^{2})}\left(mu-v\right)],$$ (6)

$$\begin{array}{l} u\frac{\partial T}{\partial x}+v\frac{\partial T}{\partial y}+w\frac{\partial T}{\partial z}=\upsilon_{nf}\frac{\partial^{2}T}{\partial z^{2}}-\lambda_{E}\left[\begin{array}{l} u^{2}\frac{\partial^{2}T}{\partial x^{2}}+v^{2}\frac{\partial^{2}T}{\partial y^{2}}+w^{2}\frac{\partial^{2}T}{\partial z^{2}}+2uv\frac{\partial^{2}T}{\partial x\partial y}+2vw\frac{\partial^{2}T}{\partial y\partial z}+2uw\frac{\partial^{2}T}{\partial x\partial z}\\ +(u\frac{\partial u}{\partial x}+v\frac{\partial u}{\partial y}+w\frac{\partial u}{\partial z})\frac{\partial T}{\partial x}+(u\frac{\partial v}{\partial x}+v\frac{\partial v}{\partial y}+w\frac{\partial v}{\partial z})\frac{\partial T}{\partial y}\\ +(u\frac{\partial w}{\partial x}+v\frac{\partial w}{\partial y}+w\frac{\partial w}{\partial z})\frac{\partial T}{\partial z} \end{array}\right]\\ -\left[\frac{1}{{\left(\rho C_{p}\right)}_{nf}}\frac{\partial q_{r}}{\partial z}\right]. \end{array}$$ (7)

The current problem’s boundary conditions are

$$u=u_{w}=c{\left(x+y\right)}^{n},\;v=v_{w}=c{\left(x+y\right)}^{n},\;w=0,\;T=T_{w},\;at\;z=A{\left(x+y\right)}^{\frac{1-n}{2}},$$ (8)

$$u\to 0\;v\to 0\;T\to T_{\infty }\;\mathrm{as}\;z\to \infty .$$ (9)

In direction of *x*−axis, *y*−axis and *z*−axis the velocity components are designed as *u*, *v* and *w*. The nanofluid kinematics viscosity is *υ*_*nf*_, *ρ*_*nf*_ is the nanofluid density, *B*_0_ is for the magnetic field, Hall current *m*, heat relaxation parameter is denoted by *λ*_*E*_, heat capacitance for nanoliquid is designated by ${\left(\rho C_{p}\right)}_{nf}$ and the radiative heat flux is $q_{r}=-(\frac{4\sigma^{*}}{3k^{*}})(\frac{\partial T^{4}}{\partial z})$, where the Stefan-Boltzman coefficient is σ* and the mean immersion coefficient is k*. The shape of the sheet, performance of the boundary layer and types of motions are measured by the shape parameter *n*. In this study, three different cases are discussed for wall thickness parameter *n*. The wall thickness shows decrement when *n*>1, when *n* = 1 then the surface is flat and the wall thickness parameter is increased for *n*<1. It is noted that types of motions are measured by the wall thickness parameter therefore, the accelerated motion is denoted by *n*>1, and for linear motion the wall thickness parameter is *n* = 1 and for the decelerated motion the wall thickness parameter is *n*<1.

### 2.1. Nanofluid properties

The physical properties for nanofluid are given as

$$\rho_{nf}=\left(\rho_{f}(1-\phi )+\rho_{p}\phi \right),\;{\left(\rho C_{p}\right)}_{nf}={\left(\rho C_{p}\right)}_{f}\left(1-\phi \right)+\phi {\left(\rho C_{p}\right)}_{p},\;\mu_{nf}=\frac{\mu_{f}}{{\left(1-\phi \right)}^{2.5}},$$ (10)

$$\frac{\sigma_{nf}}{\sigma_{f}}=(1+\frac{3(\sigma -1)\phi }{(\sigma +2)-(\sigma -1)\phi }),\;\frac{k_{nf}}{k_{f}}=\frac{\left(1-\phi \right)+2\phi \frac{k_{s}}{k_{s}-k_{f}}\ln\frac{k_{s}+k_{f}}{2k_{f}}}{\left(1-\phi \right)+2\phi \frac{k_{f}}{k_{s}-k_{f}}\ln\frac{k_{s}+k_{f}}{2k_{f}}},$$ (11)

here *ρ*_*nf*_ is for nanofluid density, heat capacitance for nanoliquid is epitomized by (*ρC*_*p*_)_*nf*_, the *μ*_*nf*_ is the dynamic viscosity for nanoliquid and nanofluid thermal conductivity is *k*_*nf*_. Table 1. summarizes the thermophysical properties of VPO−*Fe*_3_*O*_4_ nanoliquid.

**Table 1:** Thermophysical characteristics of vacuum pump oil (VPO) and *Fe*_3_*O*_4_ nanoparticles [42].

Property	VPO	*Fe*_3_*O*_4_ (nanoparticles)
*C*_*p*_ (*J*/*kg*.*K*)	2320	670
*ρ* (*kg*/*m*^3^)	870	5810
*k* (*W*/*m*.*K*)	0.13	80
*μ* (mPa.s)	93.1	--

For the present study the similarity transformations are introduced,

$$\begin{array}{l} \xi =\sqrt{\frac{n+1}{2}\frac{c}{\upsilon_{f}}{\left(x+y\right)}^{n-1}}z,u=c{\left(x+y\right)}^{n}f^{'}(\xi ),v=c{\left(x+y\right)}^{n}g^{'}(\xi ),\\ w=-\sqrt{\frac{n+1}{2}c\upsilon_{f}{\left(x+y\right)}^{n-1}}\left[f(\xi )+g(\xi )+(\frac{n-1}{n+1})\xi (f^{'}(\xi )+g^{'}(\xi ))\right],\theta (\xi )=\frac{T-T_{\infty }}{T_{w}-T_{\infty }}, \end{array}\}$$ (12)

The equation of continuity in (4) is identically satisfied by employing the above similarity transformations in Eq (12), and the non-dimensional form of the Eqs (5–7) are given as

$$a_{0}f'''+ff^{''}+gf^{''}-(\frac{2n}{n+1})f^{{'}^{2}}-(\frac{2n}{n+1})f^{'}g^{'}-a_{2}M^{2}(\frac{2}{n+1})f^{'}-a_{2}M^{2}(\frac{2}{\left(1+m^{2})(n+1\right)})(g^{'}-mf')=0,$$ (13)

$$\begin{array}{l} a_{0}g'''+fg^{''}+gg^{''}-(\frac{2n}{n+1})g^{{'}^{2}}-(\frac{2n}{n+1})f^{'}g^{'}-a_{2}M^{2}(\frac{2}{n+1})g^{'}-a_{2}M^{2}(\frac{2}{\left(1+m^{2})(n+1\right)})(mf^{'}-g')=0, \end{array}$$ (14)

$$\frac{1}{\mathrm{Pr}}(a_{1}+\frac{4}{3}a_{3}Nr)\theta^{''}+f\theta^{'}+g\theta^{'}+\delta [\begin{array}{l} \frac{{\left(n-1\right)}^{2}}{2(n+1)}\{\xi^{2}f^{'}{}^{2}\theta^{''}+\xi^{2}g^{'}{}^{2}\theta^{''}-\xi f^{''}g^{'}\theta^{'}-\xi g^{''}g^{'}\theta^{'}+\xi^{2}f^{''}g^{'}\theta^{'}+\xi^{2}g^{''}g^{'}\theta^{'}\}\\ -\frac{\left(n-1\right)}{2}(gg^{'}\theta^{'}+fg^{'}\theta^{'}+ff^{'}\theta^{'}+f^{'}g\theta^{'})+\frac{\left(n+1\right)}{2}(g^{2}\theta^{''}+f^{2}\theta^{''}+fg\theta^{''}) \end{array}]=0.$$ (15)

Now the transformed boundary conditions are

$$f^{'}(\beta )=1,\;g^{'}(\beta )=1,\;f(\beta )=-\frac{2(n-1)\beta }{\left(n+1\right)},\;g(\beta )=0,\;\theta (\beta )=1\;\mathrm{when}\;\xi =\beta ,$$ (16)

$$f^{'}(\xi )\to 0,\;g^{'}(\xi )\to 0,\;\theta (\xi )\to 0,\;\mathrm{when}\;\xi \to \infty ,$$ (17)

here the similarity variable is *ξ*, *f*’(*ζ*) is the dimensionless velocity in *x*−direction, in *y*−direction the dimensionless nanofluid velocity is *g*’(*ζ*), the dimensionless temperature is *θ*(*ζ*) and the wall thickness parameter is *β*. The dimensionless nanofluid constants are denoted by the $a_{0}=\frac{1}{{\left(1-\phi \right)}^{2.5}(1-\phi +\frac{\rho_{s}}{\rho_{f}}\phi )}$, $a_{1}=\frac{k_{nf}}{k_{f}}\frac{1}{{\left(1-\phi \right)}^{2.5}(1-\phi +\frac{{\left(\rho c_{p}\right)}_{s}}{{\left(\rho c_{p}\right)}_{f}}\phi )}$, $a_{2}=\frac{1+\frac{3(\sigma_{s}-2\sigma_{f})}{(\sigma_{s}+2\sigma_{f})-(\sigma_{s}-\sigma_{f})\phi }}{\left(1-\phi +\frac{{\left(\rho c_{p}\right)}_{s}}{{\left(\rho c_{p}\right)}_{f}}\phi \right)}$ and $a_{3}=(\frac{\frac{(1-\phi )+2\phi \frac{k_{s}}{k_{s}-k_{f}}\ln\frac{k_{s}+k_{f}}{2k_{f}}}{(1-\phi )+2\phi \frac{k_{f}}{k_{s}-k_{f}}\ln\frac{k_{s}+k_{f}}{2k_{f}}}}{\left(1-\phi +\frac{{\left(\rho c_{p}\right)}_{s}}{{\left(\rho c_{p}\right)}_{f}}\phi \right)})$ respectively. The shape parameter is *n*, the dimensionless form of magnetic field parameter is $M^{2}=\frac{\sigma_{f}B_{0}^{2}}{\rho_{f}c{\left(x+y\right)}^{n-1}}$, Hall current is *m*, $\mathrm{Pr}=\frac{\upsilon_{f}\rho C_{p}}{k_{f}}$ is used for Prandtl number, $Nr=\frac{4\sigma^{*}T_{\infty }^{3}}{k^{*}k_{nf}}$ is the thermal radiation parameter in dimensionless form and the thermal relaxation time parameter is symbolized by the $\delta =\lambda_{E}c{\left(x+y\right)}^{n-1}$.

Skin friction coefficients in *x*−axis, *y*−axis and Nusselt number are quantified as:

$$C_{fx}=\frac{\tau_{wx}}{\rho_{f}u_{w}^{2}},\;C_{fy}=\frac{\tau_{wy}}{\rho_{f}v_{w}^{2}},\;Nu_{x}=\frac{xq_{w}}{k_{f}(T_{w}-T_{\infty })},$$ (18)

where for *x*−direction and *y*−direction, the surface shear stresses *τ*_*wx*_, *τ*_*wy*_ and the wall flux temperature *q*_*w*_ are defined as

$$\tau_{wx}=\mu_{nf}{\frac{\partial u}{\partial z}|}_{z=0},\tau_{wy}=\mu_{nf}{\frac{\partial v}{\partial z}|}_{z=0},q_{w}=k_{nf}{\frac{\partial T}{\partial z}|}_{z=0},$$ (19)

the skin friction coefficients and Nusselt number in the dimensionless form are

Cfx(Rex)12=1(1−ϕ)2.5f''(0),Cfy(Rey)12=1β32(1−ϕ)2.5g''(0),Nux(Rex)−12=−knfkf1(1−ϕ)2.5θ′(0)}, (20)

here local Reynolds number is signified by (Rex)12=xcυf and (Rey)12=ycυf.

## 3. Solution of the problem

The linear operator and initial guesses for the current study are defined below

$$\begin{array}{l} f_{0}(\zeta )=-\frac{e^{-\zeta }(-e^{-\zeta }+e^{\beta }-e^{\zeta }n+e^{\beta }n-2e^{\zeta }\beta +2e^{\zeta }\beta n)}{1+n},G_{0}(\zeta )=e^{-\zeta }(e^{\zeta }-e^{\beta }),\\ \theta_{0}(\zeta )=e^{-\zeta +\beta }, \end{array}\},$$ (21)

$$L_{f}=f'''-f',L_{g}=g''-g,L_{\theta }={\theta^{\prime }}^{\prime }-\theta \},$$ (22)

then

$$\begin{array}{l} L_{F}[C_{1}+C_{2}\exp(\zeta )+C_{3}\exp(-\zeta )]=0,L_{g}[C_{4}\exp(\zeta )+C_{5}\exp(-\zeta )=0,\\ L_{\theta }[C_{6}\exp(\zeta )+C_{7}\exp(-\zeta )]=0 \end{array}\}.$$ (23)

And *C*_*i*_(*i* = 1−7) are the arbitrary constant.

### 3.1. Zeroth order deformation problem

The current model’s zero-order deformation is

$$(1-q)L_{f}[f(\zeta ,q)-f_{0}(\zeta )]=qh_{f}N_{f}[f(\zeta ,q),g(\zeta ,q)],$$ (24)

$$(1-q)L_{g}[g(\zeta ,q)-g_{0}(\zeta )]=qh_{g}N_{g}[f(\zeta ,q),g(\zeta ,q)],$$ (25)

$$(1-q)L_{\theta }[\theta (\zeta ,q)-\theta_{0}(\zeta )]=qh_{\theta }N_{\theta }[f(\zeta ,q),g(\zeta ,q),,\theta (\zeta ,q)].$$ (26)

Here q is the embedding parameter, and *h*_*f*_, *h*_*g*_ and *h*_*θ*_ are the nonzero auxiliary parameters. The nonlinear operator is denoted by *N*_*f*_, *N*_*g*_ and *N*_*θ*_ are quantified as

$$\begin{array}{l} N_{f}[f(\zeta ,q),g(\zeta ,q)]=a_{0}\frac{\partial^{3}f(\zeta ,q)}{\partial \zeta^{3}}+f(\zeta ,q)\frac{\partial^{2}f(\zeta ,q)}{\partial \zeta^{2}}+g(\zeta ,q)\frac{\partial^{2}f(\zeta ,q)}{\partial \zeta^{2}}-(\frac{2n}{n+1}){\left(\frac{\partial f(\zeta ,q)}{\partial \zeta }\right)}^{2}\\ -(\frac{2n}{n+1})\frac{\partial f(\zeta ,q)}{\partial \zeta }\frac{\partial g(\zeta ,q)}{\partial \zeta }-(\frac{2a_{2}M^{2}}{n+1})\frac{\partial f(\zeta ,q)}{\partial \zeta }-(\frac{2a_{2}M^{2}}{n+1})(\frac{2}{\left(1+m^{2})(n+1\right)})(\frac{\partial g(\zeta ,q)}{\partial \zeta }-m\frac{\partial f(\zeta ,q)}{\partial \zeta }), \end{array}$$ (27)

$$\begin{array}{l} N_{g}[f(\zeta ,q),g(\zeta ,q)]=a_{0}\frac{\partial^{3}g(\zeta ,q)}{\partial \zeta^{3}}+f(\zeta ,q)\frac{\partial^{2}g(\zeta ,q)}{\partial \zeta^{2}}+g(\zeta ,q)\frac{\partial^{2}g(\zeta ,q)}{\partial \zeta^{2}}-(\frac{2n}{n+1}){\left(\frac{\partial g(\zeta ,q)}{\partial \zeta }\right)}^{2}\\ -(\frac{2n}{n+1})\frac{\partial f(\zeta ,q)}{\partial \zeta }\frac{\partial g(\zeta ,q)}{\partial \zeta }-(\frac{2a_{2}M^{2}}{n+1})\frac{\partial g(\zeta ,q)}{\partial \zeta }-(\frac{2a_{2}M^{2}}{n+1})(\frac{2}{\left(1+m^{2})(n+1\right)})(m\frac{\partial f(\zeta ,q)}{\partial \zeta }-\frac{\partial g(\zeta ,q)}{\partial \zeta }), \end{array}$$ (28)

$$\begin{array}{l} N_{\theta }[f(\zeta ,q),g(\zeta ,q),\theta (\zeta ,q)]=\frac{1}{\mathrm{Pr}}(a_{1}+\frac{4}{3}a_{3}Nr)\frac{\partial^{2}\theta (\zeta ,q)}{\partial \zeta^{2}}+f(\zeta ,q)\frac{\partial \theta (\zeta ,q)}{\partial \zeta }+g(\zeta ,q)\frac{\partial \theta (\zeta ,q)}{\partial \zeta }\\ +\delta \left[\begin{array}{l} \frac{{\left(n-1\right)}^{2}}{2(n+1)}\left\{\begin{array}{l} \xi^{2}{\left(\frac{\partial f(\zeta ,q)}{\partial \zeta }\right)}^{2}\frac{\partial^{2}\theta (\zeta ,q)}{\partial \zeta^{2}}+\xi^{2}{\left(\frac{\partial g(\zeta ,q)}{\partial \zeta }\right)}^{2}\frac{\partial^{2}\theta (\zeta ,q)}{\partial \zeta^{2}}-\xi \frac{\partial^{2}f(\zeta ,q)}{\partial \zeta^{2}}\frac{\partial g(\zeta ,q)}{\partial \zeta }\frac{\partial \theta (\zeta ,q)}{\partial \zeta }\\ -\xi \frac{\partial^{2}g(\zeta ,q)}{\partial \zeta^{2}}\frac{\partial g(\zeta ,q)}{\partial \zeta }\frac{\partial \theta (\zeta ,q)}{\partial \zeta }+\xi^{2}\frac{\partial^{2}f(\zeta ,q)}{\partial \zeta^{2}}\frac{\partial g(\zeta ,q)}{\partial \zeta }\frac{\partial \theta (\zeta ,q)}{\partial \zeta }\\ +\xi^{2}\frac{\partial^{2}g(\zeta ,q)}{\partial \zeta^{2}}\frac{\partial g(\zeta ,q)}{\partial \zeta }\frac{\partial \theta (\zeta ,q)}{\partial \zeta } \end{array}\right\}\\ -\frac{\left(n-1\right)}{2}\left(\begin{array}{l} g(\zeta ,q)\frac{\partial g(\zeta ,q)}{\partial \zeta }\frac{\partial \theta (\zeta ,q)}{\partial \zeta }+f(\zeta ,q)\frac{\partial g(\zeta ,q)}{\partial \zeta }\frac{\partial \theta (\zeta ,q)}{\partial \zeta }+f(\zeta ,q)\frac{\partial f(\zeta ,q)}{\partial \zeta }\frac{\partial \theta (\zeta ,q)}{\partial \zeta }\\ +g(\zeta ,q)\frac{\partial f(\zeta ,q)}{\partial \zeta }\frac{\partial \theta (\zeta ,q)}{\partial \zeta } \end{array}\right)\\ +\frac{\left(n+1\right)}{2}({\left(g(\zeta ,q)\right)}^{2}\frac{\partial^{2}\theta (\zeta ,q)}{\partial \zeta^{2}}+{\left(f(\zeta ,q)\right)}^{2}\frac{\partial^{2}\theta (\zeta ,q)}{\partial \zeta^{2}}+f(\zeta ,q)g(\zeta ,q)\frac{\partial^{2}\theta (\zeta ,q)}{\partial \zeta^{2}}) \end{array}\right], \end{array}$$ (29)

$$f^{'}(0,q)=1,\;\mathrm{and}\;f^{'}(\infty ,q)=0,$$ (30)

$$g^{'}(0,q)=1,\;\mathrm{and}\;g^{'}(\infty ,q)=0,$$ (31)

θ(0,q)=1,andθ(∞,q)=0, (32)

For *q* = 0 and *q* = 1 then the Eqs (24–26) become as

$$q=0\Rightarrow f(\zeta ,0)=f_{0}(\zeta )\;\mathrm{and}\;q=1\Rightarrow f(\zeta ,1)=f(\zeta ),$$ (33)

$$q=0\Rightarrow g(\zeta ,0)=g_{0}(\zeta )\;\mathrm{and}\;q=1\Rightarrow g(\zeta ,1)=g(\zeta ),$$ (34)

$$q=0\Rightarrow \theta (\zeta ,0)=\theta_{0}(\zeta )\;\mathrm{and}\;q=1\Rightarrow \theta (\zeta ,1)=\theta (\zeta ),$$ (35)

The Taylor series expansion is applied on Eqs (33–35), it is obtained that

$$f(\zeta ,q)=f_{0}(\zeta )+\sum_{m=1}^{\infty }f_{m}(\zeta )q^{m},\;f_{m}(\zeta )=\frac{1}{m!}\frac{\partial^{m}f(\zeta ,q)}{\partial \zeta^{m}}{|}_{q=0},$$ (36)

$$g(\zeta ,q)=g_{0}(\zeta )+\sum_{m=1}^{\infty }g_{m}(\zeta )q^{m},\;g_{m}(\zeta )=\frac{1}{m!}\frac{\partial^{m}g(\zeta ,q)}{\partial \zeta^{m}}{|}_{q=0},$$ (37)

$$\theta (\zeta ,q)=\theta_{0}(\zeta )+\sum_{m=1}^{\infty }\theta_{m}(\zeta )q^{m},\;\theta_{m}(\zeta )=\frac{1}{m!}\frac{\partial^{m}\theta (\zeta ,q)}{\partial \zeta^{m}}{|}_{q=0},$$ (38)

The convergence of the series is attained when *q* = 1 in Eqs (36–38)

$$f(\zeta )=f_{0}(\zeta )+\sum_{m=1}^{\infty }f_{m}(\zeta ),$$ (39)

$$g(\zeta )=g_{0}(\zeta )+\sum_{m=1}^{\infty }g_{m}(\zeta ),$$ (40)

$$\theta (\zeta )=\theta_{0}(\zeta )+\sum_{m=1}^{\infty }\theta_{m}(\zeta ),$$ (41)

### 3.2. m^th^ order deformation problem

The m^*th*^ order form of the problem is

$$L_{f}[f_{m}(\zeta )-\eta_{m}f_{m-1}(\zeta )]=h_{F}R_{m}^{f}m(\zeta ),$$ (42)

$$L_{g}[g_{m}(\zeta )-\eta_{m}g_{m-1}(\zeta )]=h_{G}R_{m}^{g}(\zeta ),$$ (43)

$$L_{\theta }[\theta_{m}(\zeta )-\eta_{m}\theta_{m-1}(\zeta )]=h_{\theta }R_{m}^{\theta }m(\zeta ),$$ (44)

$$f_{m}(0)=0,f_{m}(\infty )=0,$$ (45)

$$g_{m}(0)=0,g_{m}(\infty )=0,$$ (46)

$$\theta_{m}(0)=0,\theta_{m}(\infty )=0,$$ (47)

The $R_{m}^{f}m(\zeta )$, $R_{m}^{g}m(\zeta )$ and $R_{m}^{\theta }m(\zeta )$ are defined as

$$\begin{array}{l} R_{m}^{f}(\zeta )=a_{0}f_{m-1}^{'''}+{\sum_{k=0}^{m-1}f}_{m-1-k}f_{k}^{''}+{\sum_{k=0}^{m-1}g}_{m-1-k}f_{k}^{''}-(\frac{2n}{n+1})\sum_{k=0}^{m-1}f_{m-1-k}^{'}f_{k}^{'}-(\frac{2n}{n+1})\sum_{k=0}^{m-1}f_{m-1-k}^{'}g_{k}^{'}-(\frac{2a_{2}M^{2}}{n+1})f_{m-1}^{'}\\ -(\frac{2a_{2}M^{2}}{\left(1+m^{2})(n+1\right)})(g_{m-1}^{'}-mf_{m-1}^{'}), \end{array}$$ (48)

$$\begin{array}{l} R_{m}^{g}(\zeta )=a_{0}g_{m-1}^{'''}+{\sum_{k=0}^{m-1}f}_{m-1-k}g_{k}^{''}+{\sum_{k=0}^{m-1}g}_{m-1-k}g_{k}^{''}-(\frac{2n}{n+1})\sum_{k=0}^{m-1}g_{m-1-k}^{'}g_{k}^{'}-(\frac{2n}{n+1})\sum_{k=0}^{m-1}f_{m-1-k}^{'}g_{k}^{'}-(\frac{2a_{2}M^{2}}{n+1})g_{m-1}^{'}\\ -(\frac{2a_{2}M^{2}}{\left(1+m^{2})(n+1\right)})(mf_{m-1}^{'}-g_{m-1}^{'}), \end{array}$$ (49)

$$\begin{array}{l} R_{m}^{\theta }(\zeta )=\frac{1}{\mathrm{Pr}}(a_{1}+\frac{4}{3}a_{3}Nr)\theta_{m-1}^{''}+\sum_{k=0}^{m-1}f_{m-1-k}\theta_{k}^{'}+\sum_{k=0}^{m-1}g_{m-1-k}\theta_{k}^{'}\\ +\delta \left[\begin{array}{l} \frac{{\left(n-1\right)}^{2}}{2(n+1)}\left\{\begin{array}{l} \xi^{2}\sum_{k=0}^{m}\left(\sum_{1=0}^{k}f^{'}f_{k-1}^{'}\right)\theta_{m-k}^{''}+\xi^{2}\sum_{k=0}^{m}\left(\sum_{1=0}^{k}g^{'}g_{k-1}^{'}\right)\theta_{m-k}^{''}-\xi \sum_{k=0}^{m}\left(\sum_{1=0}^{k}f^{''}g_{k-1}^{'}\right)\theta_{m-k}^{'}-\xi \\ \sum_{k=0}^{m}\left(\sum_{1=0}^{k}g^{''}g_{k-1}^{'}\right)\theta_{m-k}^{'}+\xi^{2}\sum_{k=0}^{m}\left(\sum_{1=0}^{k}f^{''}g_{k-1}^{'}\right)\theta_{m-k}^{'}+\xi^{2}\sum_{k=0}^{m}\left(\sum_{1=0}^{k}g^{''}g_{k-1}^{'}\right)\theta_{m-k}^{'} \end{array}\right\}\\ -\frac{\left(n-1\right)}{2}(\sum_{k=0}^{m}\left(\sum_{1=0}^{k}gg_{k-1}^{'}\right)\theta_{m-k}^{'}+\sum_{k=0}^{m}\left(\sum_{1=0}^{k}fg_{k-1}^{'}\right)\theta_{m-k}^{'}+\sum_{k=0}^{m}\left(\sum_{1=0}^{k}ff_{k-1}^{'}\right)\theta_{m-k}^{'}+\sum_{k=0}^{m}\left(\sum_{1=0}^{k}f^{'}g_{k-1}\right)\theta_{m-k}^{'})\\ +\frac{\left(n+1\right)}{2}(\sum_{k=0}^{m}\left(\sum_{1=0}^{k}gg_{k-1}\right)\theta_{m-k}^{''}+\sum_{k=0}^{m}\left(\sum_{1=0}^{k}ff_{k-1}\right)\theta_{m-k}^{''}f^{2}\theta^{''}+\sum_{k=0}^{m}\left(\sum_{1=0}^{k}fg_{k-1}\right)\theta_{m-k}^{''}) \end{array}\right], \end{array}$$ (50)

$$\eta_{m}=\left\{ \begin{array}{l} 0,\;m\leq 1\\ 1,\;m>1. \end{array}\right.$$ (51)

The general solution of the present investigation is attained by the use of the particular solution

$$f_{m}(\zeta )=f_{m}^{*}(\zeta )+C_{1}+C_{2}\exp(\zeta )+C_{3}\exp(-\zeta ),$$ (52)

$$g_{m}(\zeta )=g_{m}^{*}(\zeta )+C_{4}\exp(\zeta )+C_{5}\exp(-\zeta ),$$ (53)

$$\theta_{m}(\zeta )=\theta_{m}^{*}(\zeta )+C_{6}\exp(\zeta )+C_{7}\exp(-\zeta ),$$ (54)

## 4. Validation

Table 2 is made for the comparison of the current fallouts with the previously published results. We compared our new results to Rashidi et al. [43] and Dogonchi and Ganji [44] previously published results to validate the current model. From this Table 2, it is observed that an excellent agreement is obtained with the previous published results of Rashidi et al. [43] and Dogonchi and Ganji [44].

**Table 2:** Comparison between present and Rashidi et al. [43] & Dogonchi and Ganji [44] results for −*f*″(0) and −*θ*′(0).

*Nr*	Published results [43] −*f*″(0)	Published results [43] −*θ*′(0)	Published results [44] −*f*″(0)	Published results [44] −*θ*′(0)	Present results −*f*″(0)	Present results −*θ*′(0)
1.00	1.41599846	0.71647710	1.41600362	0.71649390	1.416006543	0.71649345
2.00	1.44214325	0.88544087	1.44214350	0.88544181	1.442143896	0.88544975
3.00	1.45336332	0.96827471	1.45336336	0.96827499	1.453373132	0.96829043
*ϕ*						
0.10	1.44726274	0.93553568	1.44726283	0.93553609	1.447265649	0.93554370
0.12	1.44214325	0.88544087	1.44214350	0.88544181	1.442107561	0.88544538
0.15	1.42985157	0.81376547	1.42985251	0.81376880	1.429855785	0.81376876

## 5. Results and discussion

This portion looked at the problem’s physical aspects. The analytical solution of the coupled non-linear ODEs (13–15) along with boundary conditions (16–17) are resolved by the assistance of the homotopy analysis technique in MATHEMATICA 10. The analysis of the involved parameters against skin friction coefficient *Cf*_*x*_, *Cf*_*y*_ and Nusselt number *Nu*_*x*_ are described in a tabular form. Also, the outcomes of distinct parameters such as Hall current *m*, magnetic field parameter *M*, shape parameter *n*, nanoparticles volume fraction *ϕ*, radiation parameter *Nr* and thermal relaxation time parameter *δ* over the velocities in *x*−direction, *y*−direction and temperature profiles of the nanofluid have been examined and discussed in a graphical form. In graphical discussion, the ranges of all effective parameter are fixed and one parameter varies to plot their respective graph. For different parameters the ranges are Pr = 1000, *m* = 0.10, *n* = 1.00, *M* = 2.50, *δ* = 0.40, *β* = 1.00, *Nr* = 0.20 and *ϕ* = 0.01 to *ϕ* = 0.04. It is noticed that the ranges of Prandtl number for Engine oil and Vacuum pump oil and different lubricants etc is from Pr = 600 to Pr = 6000, so therefore we have considered Pr = 1000. And the *ϕ* is the nanoparticles volume fraction of the nanoliquid and its ranges is also from 0.01 to 0.04, it means that we can add 4% of nanoparticles in the base liquid. Table 3 highlighted the influence of Hall current *m*, *M*, *n*, nanoparticles volume fraction *ϕ* and wall thickness parameter *β* over the *Cf*_*x*_ and *Cf*_*y*_. It is observed from Table 3 that when shape parameter *n*, *M*, *ϕ* and *β* of the nanoliquid is enhanced then the *Cf*_*x*_ in the *x*−direction is also improved but the decrementing behavior of the *Cf*_*x*_ in *x*−direction is noted for higher estimations of *m*. Also from Table 3, it is distinguished that the *Cf*_*y*_ of the nanofluid in *y*−direction upsurges against the enhancing estimations of *ϕ* and wall thickness parameter *β* but the reverse tendency is perceived in the *Cf*_*y*_ of the nanoliquid for varying estimation of *m*, *n* and *M*. The effects of Hall current *m*, *M*, *n*, nanoparticles volume fraction *ϕ*, wall thickness parameter *β* and thermal relaxation time parameter *δ* on the *Nu*_*x*_ are exposed in Table 4. It is noticed that higher estimations of Hall current *m*, magnetic field parameter *M*, nanoparticles volume fraction *ϕ* and *β* rise the *Nu*_*x*_ of the nanofluid but the shape parameter *n*, radiation parameter *Nr* and *δ* reduced the *Nu*_*x*_ of the nanofluid.

**Table 3:** Skin friction coefficients *Cf*_*x*_ and *Cf*_*y*_ variation against *m*, *n*, *M*, *ϕ* and *β*.

*m*	*n*	*M*	*ϕ*	*β*	*Cf*_*x*_	*Cf*_*y*_
0.1					-3.543850	-4.812340
0.2					-3.542497	-4.808980
0.3					-3.541087	-4.805477
0.4					-3.539699	-4.802030
	0.2				-3.533965	-4.787761
	0.4				-3.846515	-3.584467
	0.6				-3.901506	-3.120308
	0.8				-3.948600	-2.743557
		0.3			-3.533965	-4.787761
		0.4			-3.542568	-4.766294
		0.5			-3.553628	-4.738699
		0.6			-3.567143	-4.704981
			0.01		-2.784711	-3.772681
			0.02		-2.856302	-3.869672
			0.03		-2.930483	-3.970173
			0.04		-3.007394	-4.074374
				1.0	-2.856306	-3.269677
				1.1	-2.865667	-3.342548
				1.2	-3.139135	-3.406813
				1.3	-3.429125	-3.533780

**Table 4:** Nusselt number *Nu*_*x*_ variation against *m*, *n*, *M*, *ϕ*, *Nr*, *β* and *δ*.

S	S	*M*	*ϕ*	*Nr*	*β*	*δ*	*Nu*_*x*_
0.1							-3.143508
0.2							-3.143531
0.3							-3.143556
0.4							-3.143579
	0.2						-3.143678
	0.4						-3.142813
	0.6						-2.960815
	0.8						-2.824537
		0.3					-3.143678
		0.4					-3.143730
		0.5					-3.143796
		0.6					-3.143877
			0.01				-2.477174
			0.02				-2.540855
			0.03				-2.606846
			0.04				-2.675267
				0.1			-2.562394
				0.3			-2.519443
				0.5			-2.477032
				0.7			-2.435171
					1.0		-2.540856
					1.1		-2.548537
					1.2		-2.693255
					1.3		-2.808550
						1.0	-2.130435
						1.2	-2.037614
						1.4	-1.948716
						1.6	-1.863734

### 5.1. *x*−component of velocity

**Fig 2:**
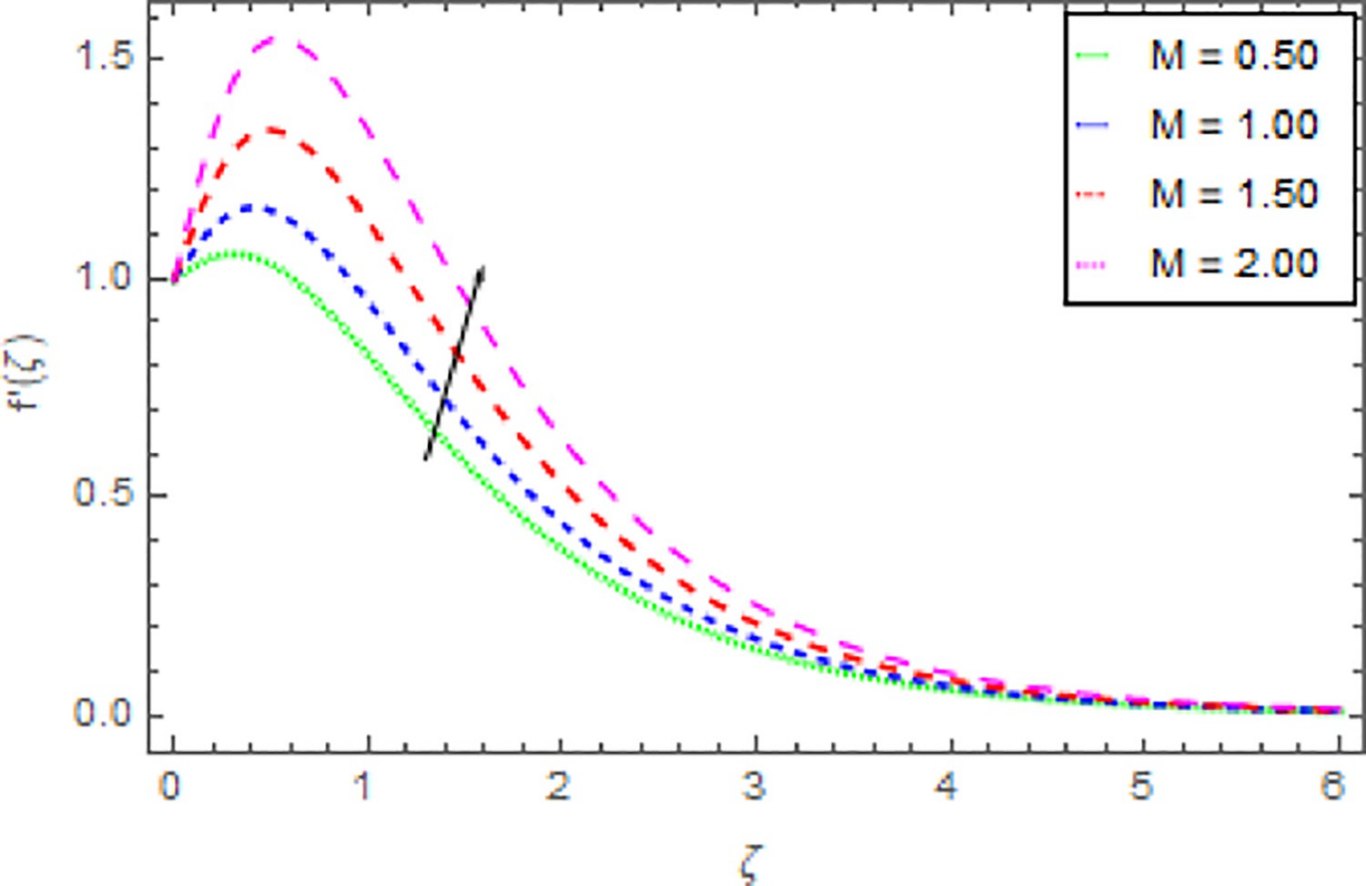
Deviation in nanofluid velocity (*x*−direction) against *M* when Pr = 1000, *m* = 0.10, *n* = 1.00, *δ* = 0.40, *β* = 1.00, *Nr* = 0.20 and *ϕ* = 0.01 to *ϕ* = 0.04.

**Fig 3:**
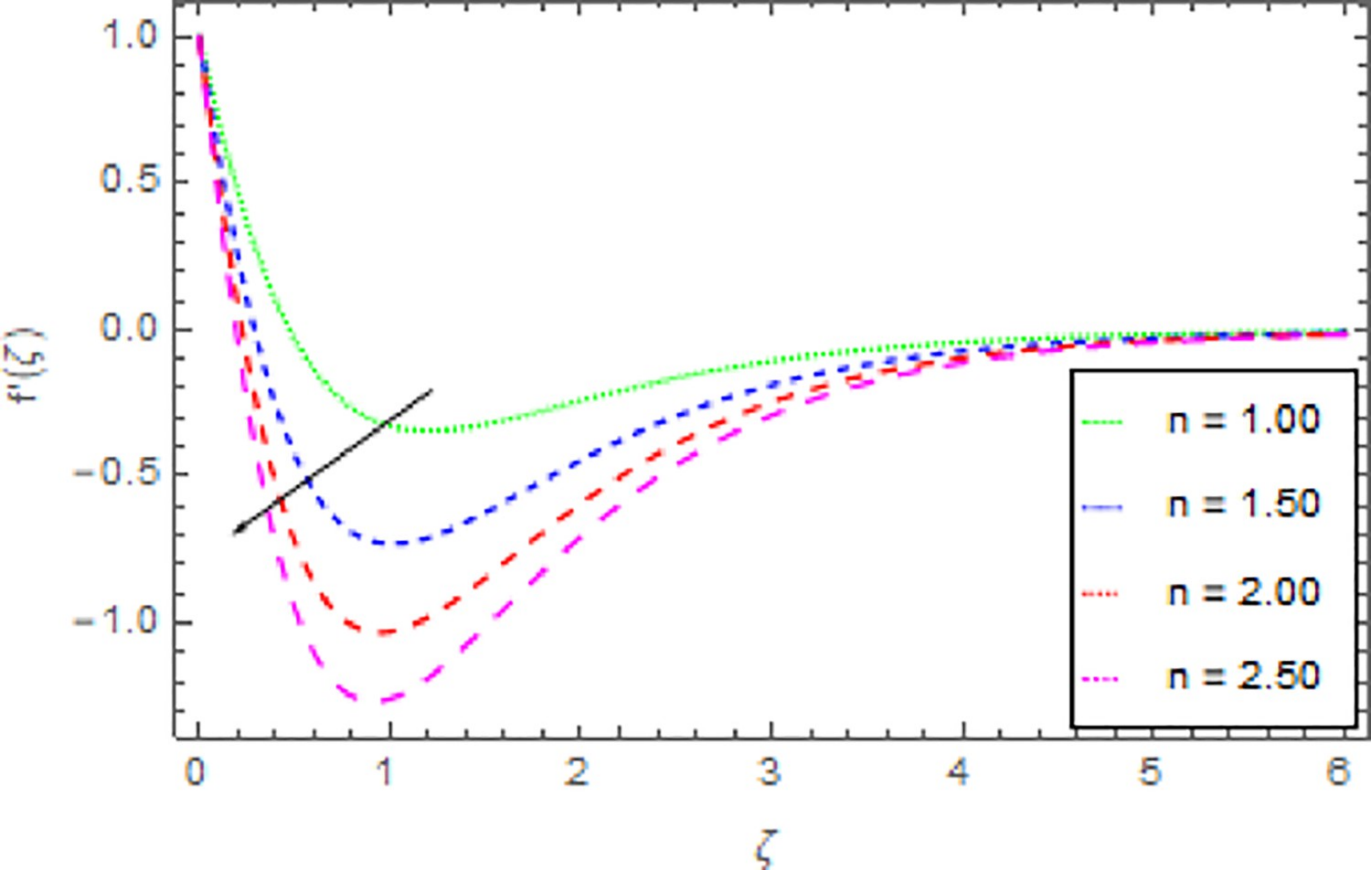
Deviation in nanofluid velocity (*x*−direction) against *n* when Pr = 1000, *m* = 0.10, *M* = 2.50, *δ* = 0.40, *β* = 1.00, *Nr* = 0.20 and *ϕ* = 0.01 to *ϕ* = 0.04.

**Fig 4:**
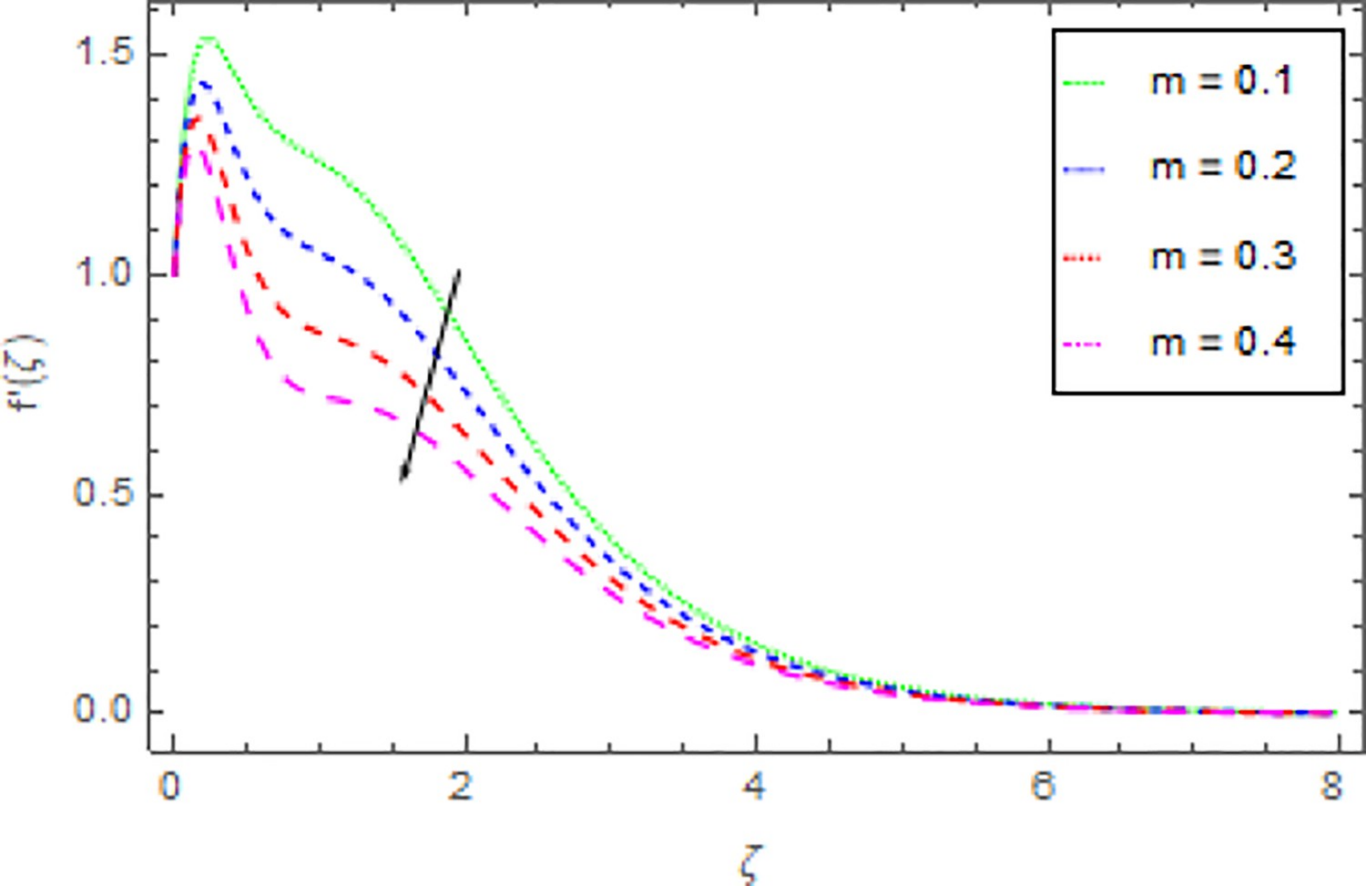
Deviation in nanofluid velocity (*x*−direction) against *m* when Pr = 1000, *n* = 1.00, *M* = 2.50, *δ* = 0.40, *β* = 1.00, *Nr* = 0.20 and *ϕ* = 0.01 to *ϕ* = 0.04.

**Fig 5:**
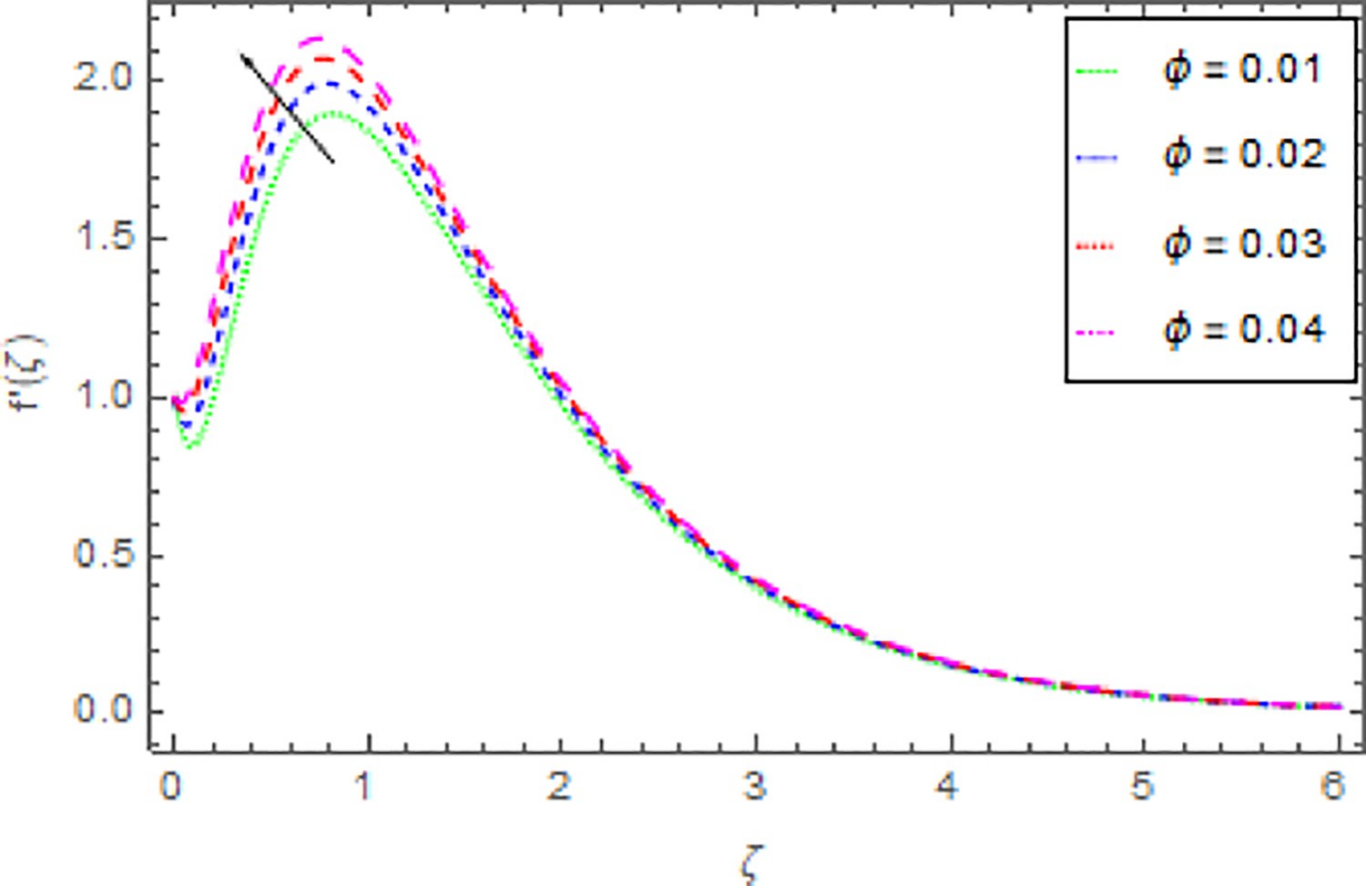
Deviation in nanofluid velocity (*x*−direction) against *ϕ* when Pr = 1000, *m* = 0.10, *n* = 1.00, *M* = 2.50, *δ* = 0.40, *β* = 1.00 and *Nr* = 0.20.

The graphical discussion of the nanofluid velocity in *x*−direction for discrete estimations of magnetic field parameter *M*, shape parameter *n*, Hall current parameter *m* and nanoparticles volume fraction *ϕ* are explained in Figs 2–5. Fig 2 represents the change of nanofluid velocity in *x*−direction for a larger estimation of *M*. It is detected that augmentation in *M* improves the nanoliquid velocity in *x*−direction. When the intensity of the magnetic field parameter is higher than the momentum boundary layer thickness shows the increasing effect therefore the velocity of the nanofluid in *x*−direction is enlarged. Fig 3 described the impression of *n* in *x*−direction on the nanoliquid velocity. From this inquiry, the reducing impact on nanoliquid velocity in *x*−direction is perceived for expanding estimations of *n*. Fig 4 explored the outcome of *m* over the nanoliquid velocity in *x*−direction. It is distinguished that the velocity of the nanoliquid in *x*−direction is decayed through the increment of *m*. The consequence of *ϕ* on the nanoliquid velocity profile in *x*−direction is established in Fig 5. It is detected that grander estimation of nanoparticles volume fraction *ϕ* enhanced the nanoliquid velocity in *x*−direction. Physically, the thermal conductivity of the nanoliquid is raised due to the intensification of *ϕ* that’s why the motion of the fluid become higher and higher.

### 5.2. *y*−component of velocity

**Fig 6:**
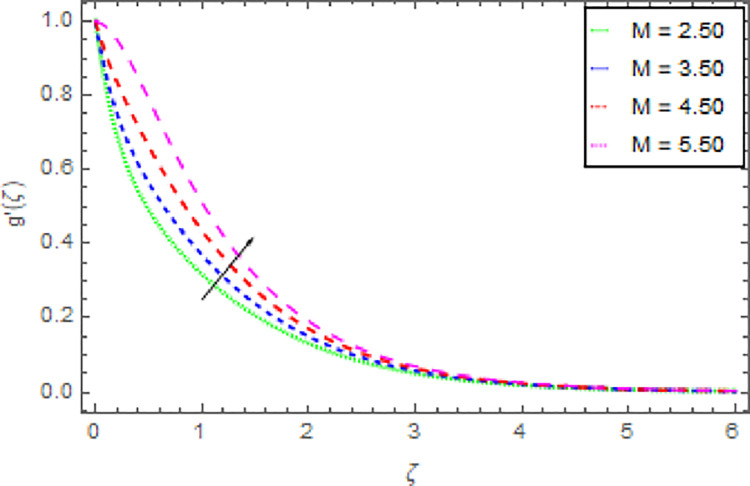
Deviation in nanofluid velocity (*y*−direction) against *M* when Pr = 1000, *m* = 0.10, *n* = 1.00, *δ* = 0.40, *β* = 1.00, *Nr* = 0.20 and *ϕ* = 0.01 to *ϕ* = 0.04.

**Fig 7:**
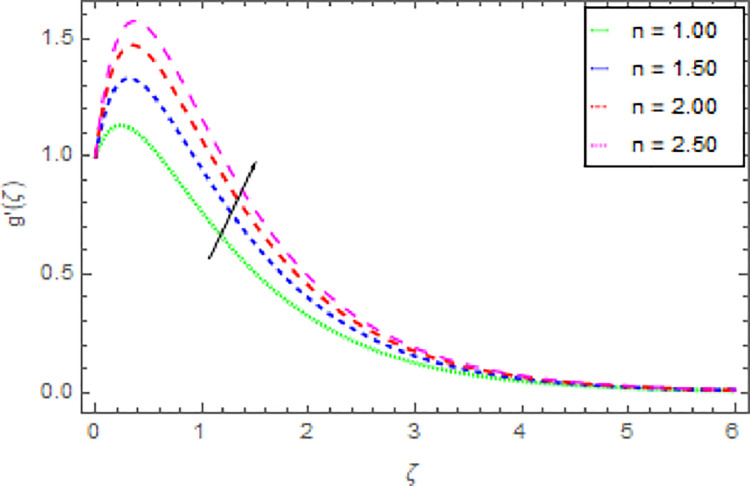
Deviation in nanofluid velocity (*y*−direction) against *n* when Pr = 1000, *m* = 0.10, *M* = 2.50, *δ* = 0.40, *β* = 1.00, *Nr* = 0.20 and *ϕ* = 0.01 to *ϕ* = 0.04.

**Fig 8:**
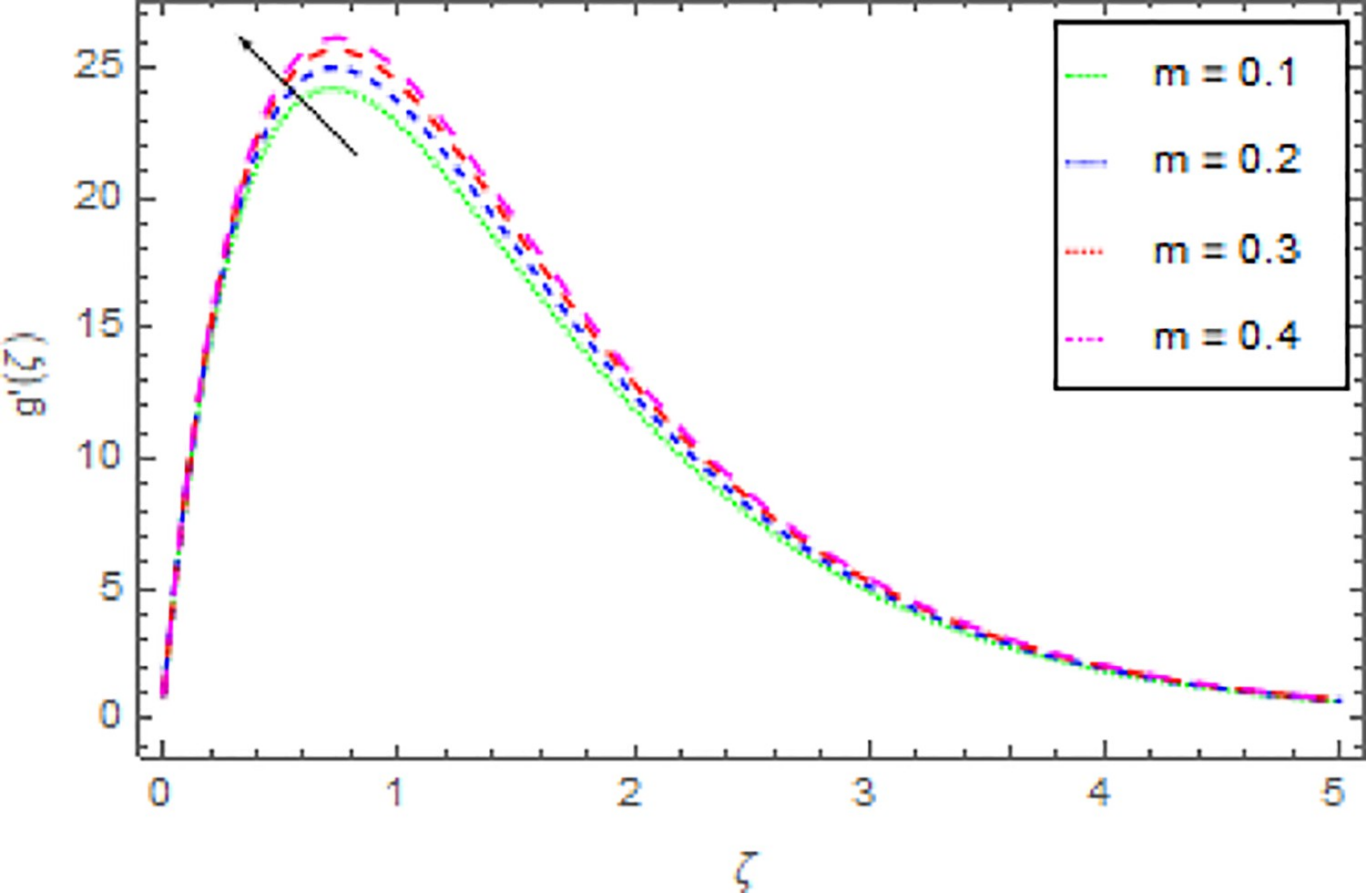
Deviation in nanofluid velocity (*y*−direction) against *m* when Pr = 1000, *n* = 1.00, *M* = 2.50, *δ* = 0.40, *β* = 1.00, *Nr* = 0.20 and *ϕ* = 0.01 to *ϕ* = 0.04.

**Fig 9:**
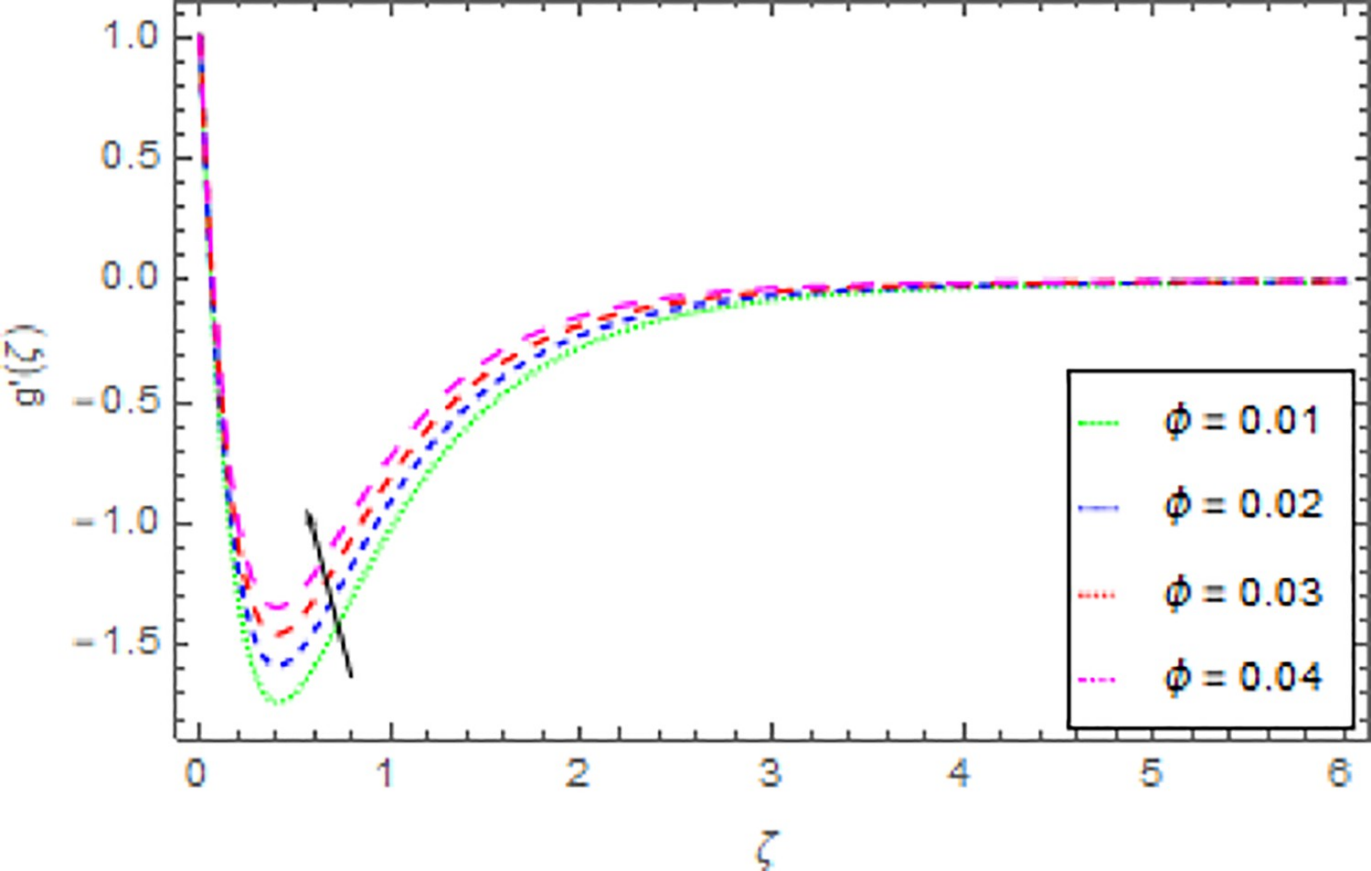
Deviation in nanofluid velocity (*y*−direction) against *ϕ* Pr = 1000, *m* = 0.10, *n* = 1.00, *M* = 2.50, *δ* = 0.40, *β* = 1.00 and *Nr* = 0.20.

Figs 6–9 are graphed to investigate the role of magnetic field parameter *M*, shape parameter *n* and nanoparticles volume fraction *ϕ* through the nanoliquid velocity in *y*−direction. The performance of the nanoliquid velocity in *y*−direction for an intensifying estimation of *M* is displayed in Fig 6. From this analysis, it is identified that the nanoliquid velocity in *y*−direction is boosted for higher estimation of *M*. Fig 7 illustrates the impression of nanoliquid velocity in *y*−direction against shape parameter *n*. It is predicted that, the varied estimation of *n* causes to enhanced the nanoliquid velocity in *y*−direction. By expanding the shape parameter *n*, the more deformation is created in the liquid that’s why the stretching velocity in *y*−direction is enhanced. The disparity of nanofluid velocity in *y*−direction against the *m* is demonstrated in Fig 8. From this Fig 8, it is reviewed that the velocity of the nanoliquid in *y*−direction is amplified for larger estimation of *m*. By expanding the effect of Hall current the momentum boundary layer thickness of the nanoliquid is augmented. Further, the enhanced Hall parameter overpowers the opposing magnetic field and accelerates the liquid velocity. Fig 9 is graphed to check the influence of *ϕ* over the velocity of the nanoliquid in *y*−direction. It is renowned that with the enrichment of *ϕ*, the velocity of the nanoliquid in *y*−direction is increased. The increment in nanoparticles volume fraction enhanced the thermal conductivity of the nanoliquid that’s why the velocity of the nanoliquid is increased.

### 5.3. Temperature profile

**Fig 10:**
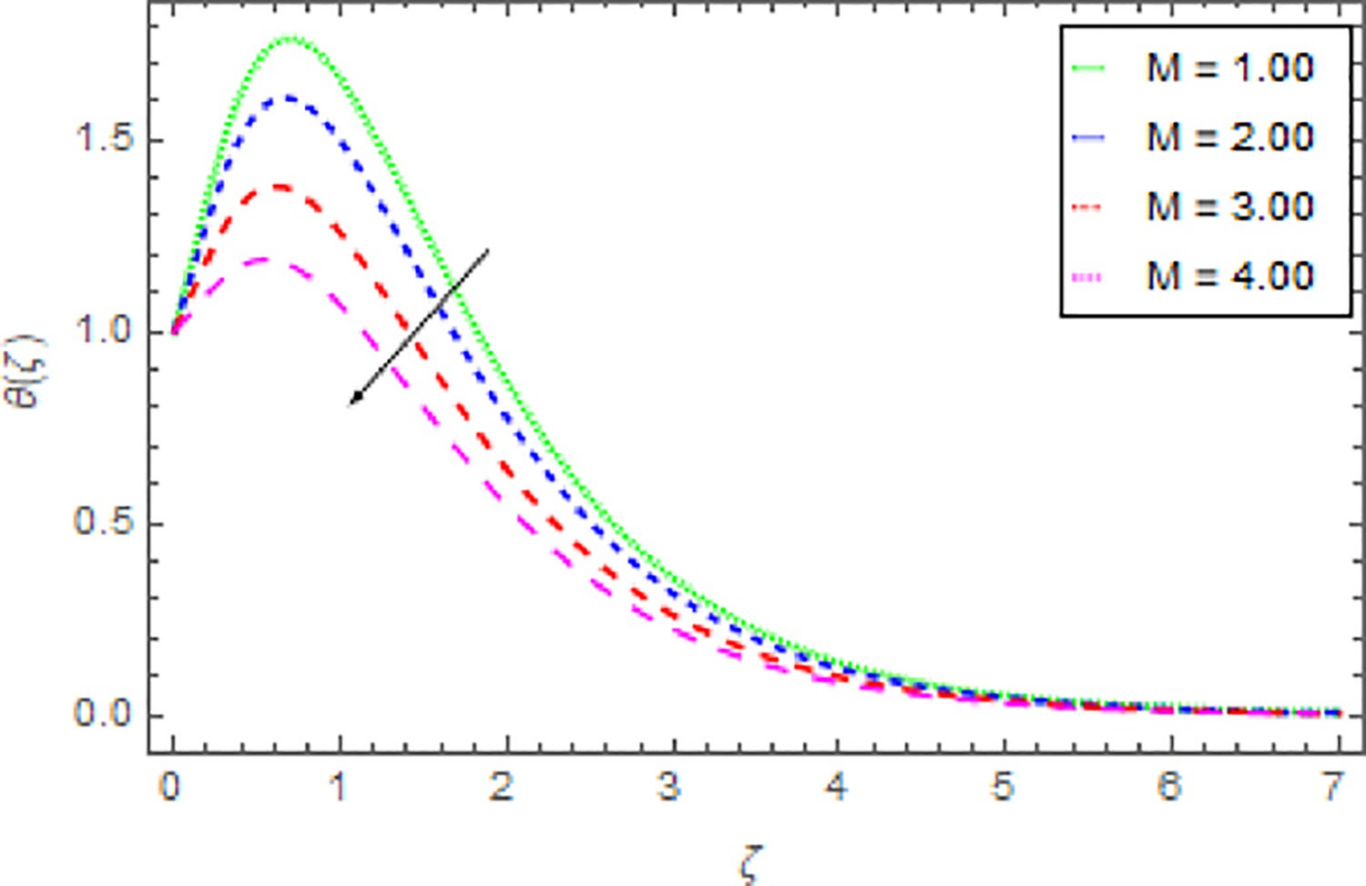
Deviation in nanofluid temperature against *M* when Pr = 1000, *m* = 0.10, *n* = 1.00, *δ* = 0.40, *β* = 1.00, *Nr* = 0.20 and *ϕ* = 0.01 to *ϕ* = 0.04.

**Fig 11:**
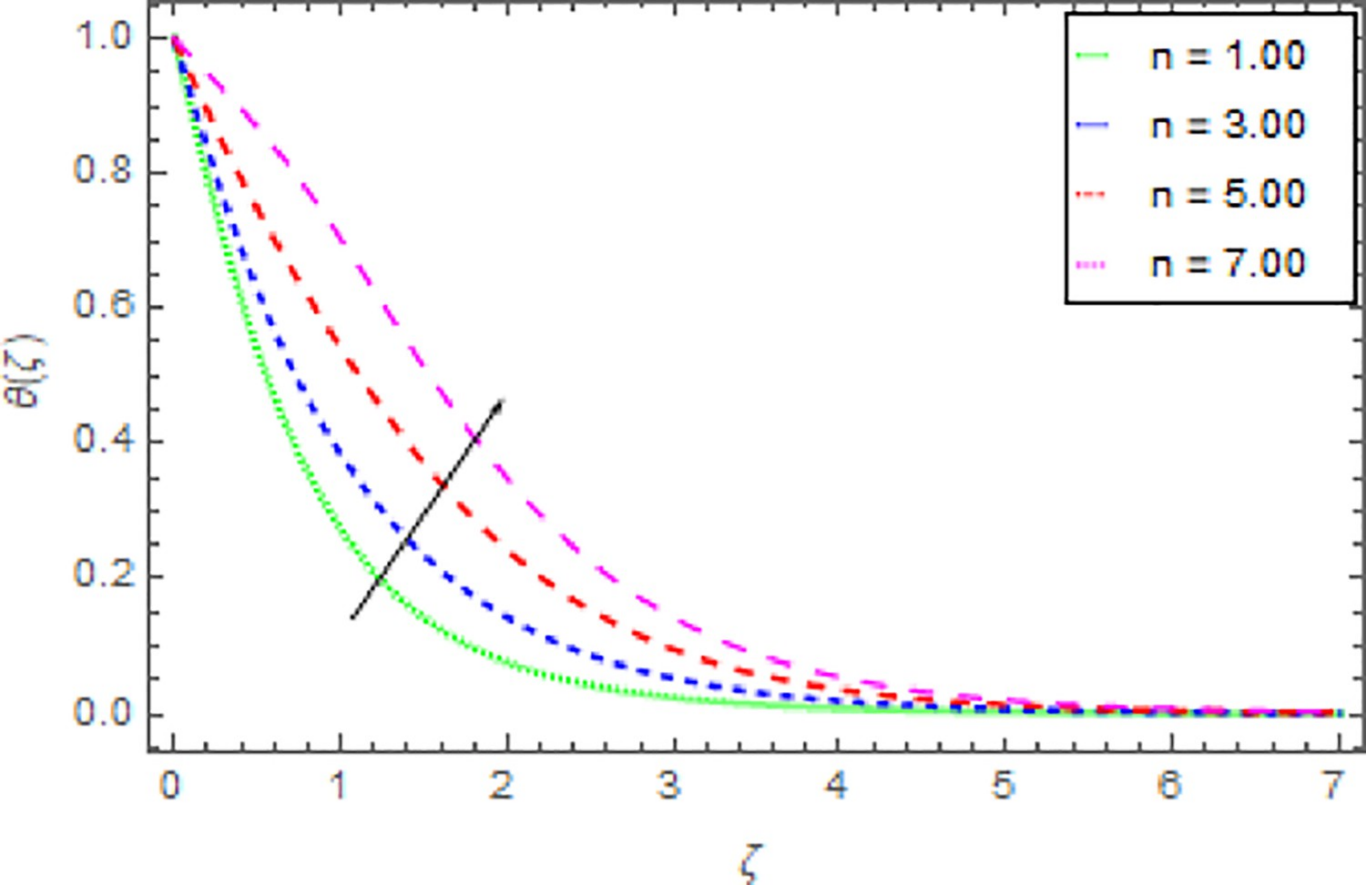
Deviation in nanofluid temperature against *n* when Pr = 1000, *m* = 0.10, *n* = 1.00, *M* = 2.50, *δ* = 0.40, *β* = 1.00, *Nr* = 0.20 and *ϕ* = 0.01 to *ϕ* = 0.04.

**Fig 12:**
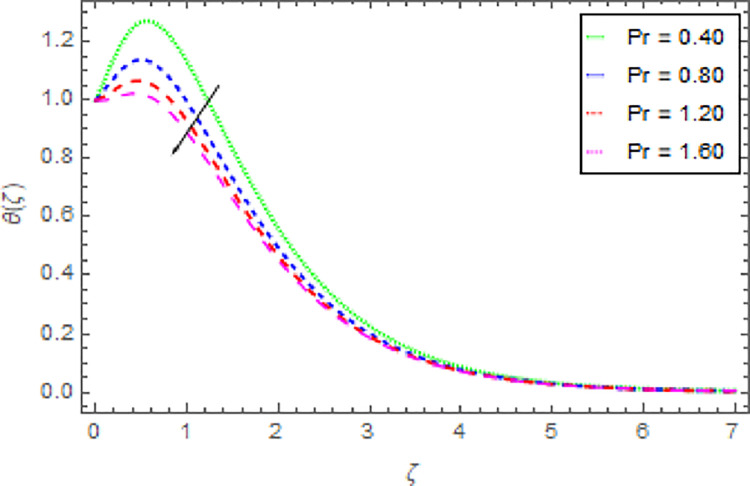
Deviation in nanofluid temperature against Pr when *m* = 0.10, *n* = 1.00, *M* = 2.50, *δ* = 0.40, *β* = 1.00, *Nr* = 0.20 and *ϕ* = 0.01 to *ϕ* = 0.04.

**Fig 13:**
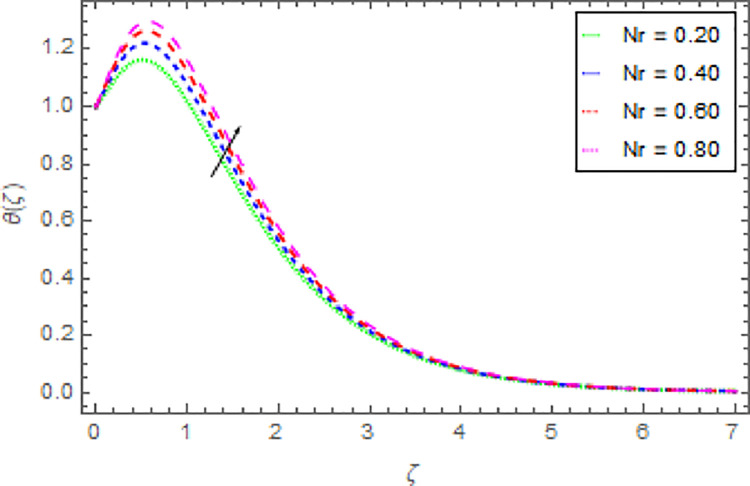
Deviation in nanofluid temperature against *Nr* Pr = 1000, *m* = 0.10, *n* = 1.00, *M* = 2.50, *δ* = 0.40, *β* = 1.00 and *ϕ* = 0.01 to *ϕ* = 0.04.

**Fig 14:**
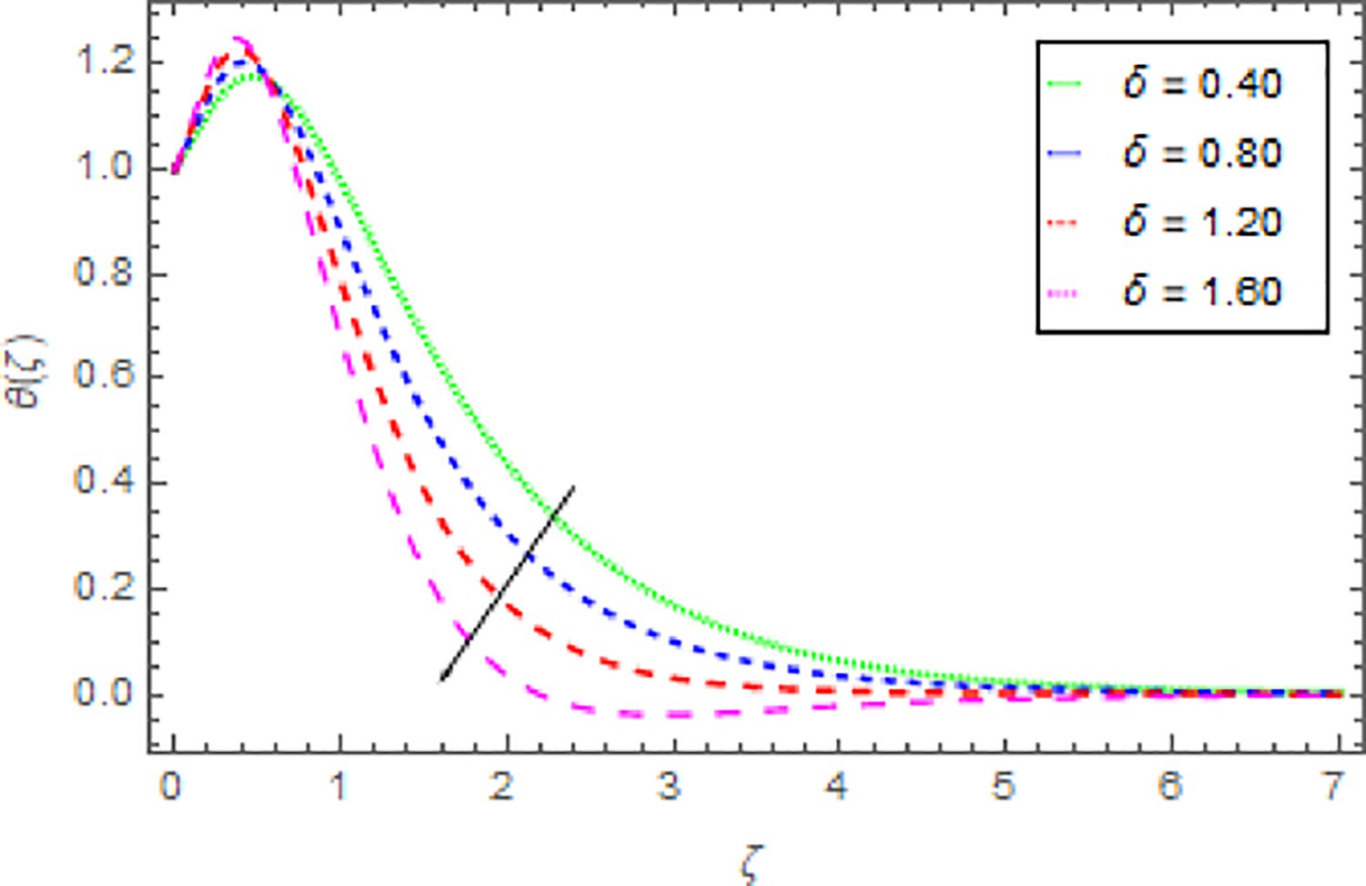
Deviation in nanofluid temperature against *δ* when Pr = 1000, *m* = 0.10, *n* = 1.00, *M* = 2.50, *β* = 1.00, *Nr* = 0.20 and *ϕ* = 0.01 to *ϕ* = 0.04.

**Fig 15:**
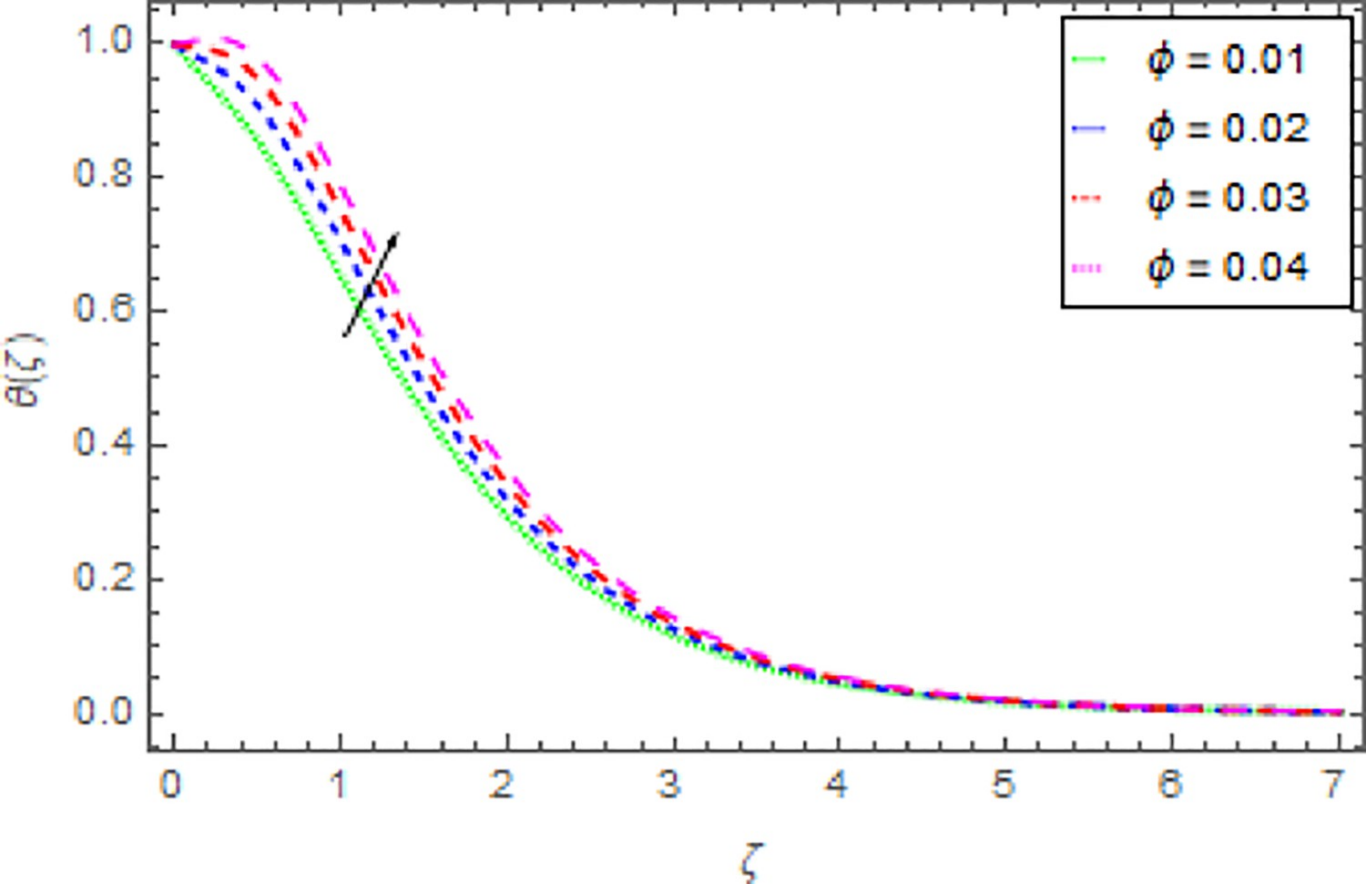
Deviation in nanofluid temperature against *ϕ* when Pr = 1000, *m* = 0.10, *n* = 1.00, *M* = 2.50, *δ* = 0.40, *β* = 1.00 and *Nr* = 0.20.

Figs 10–15 are designs for the evolution of nanofluid temperature against varying values of distinct parameters such that, the magnetic field parameter *M*, shape parameter *n*, Prandtl number Pr, radiation parameter *Nr* and thermal relaxation time parameter *δ*. The deviation in temperature of nanoliquid for rising estimation of *M* is depicted in Fig 10. From the result of this Fig, it is examined that with the enhancement of *M* the decline in nanofluid temperature is distinguished. Fig 11 exhibits the relationship between nanofluid temperature and shape parameter *n*. From this analysis, it is stated that when the shape parameter *n* of the stretched sheet is greater than the nanofluid temperature becomes higher and higher and the thermal boundary layer thickness increases. Fig 12 revealed the difference of nanofluid temperature for varying values of Pr. The diminishes in nanofluid temperature is seen in Fig 11 due to the growing estimation of the Pr. The reason is that, Pr is defined as the ratio between momentum diffusivity and thermal diffusivity. The liquid thermal conductivity is smaller when the Prandtl number becomes greater and greater and the boundary layer format is thinner. Heat diffuses out more quickly from the surface because the thickness of the thermal boundary layer is reduced. Therefore, the heat spread more rapidly from the surface and then the temperature is reduced. The fluctuation in nanoliquid temperature for upshot estimation of the radiation parameter *Nr* is deliberated in Fig 13. Larger estimations of radiation parameter *Nr* boosted the nanoliquid temperature. Fig 14 is graphed to discuss the impact of *δ* over the nanoliquid temperature. In this study, it is noted that the nanofluid temperature is declined for upgraded estimation of *δ* and the thermal boundary layer thickness diminishes. The justification for this is that as the thermal relaxation parameter is elevated, then the material’s particles needed extra time to transport heat to their nearby particles. That’s why the higher estimation of thermal relaxation parameter reduced the nanoliquid temperature. Fig 15 reflects the presence of *ϕ* on the nanoliquid temperature against the greater estimation of *ϕ*. It is scrutinized that with the escalating of *ϕ* the nanoliquid temperature is heightened. The thicker thermal boundary layer is created due to the increase of viscous forces and *ϕ* and thus the nanofluid temperature intensified.

### 5.4. Skin friction coefficient profile

**Fig 16:**
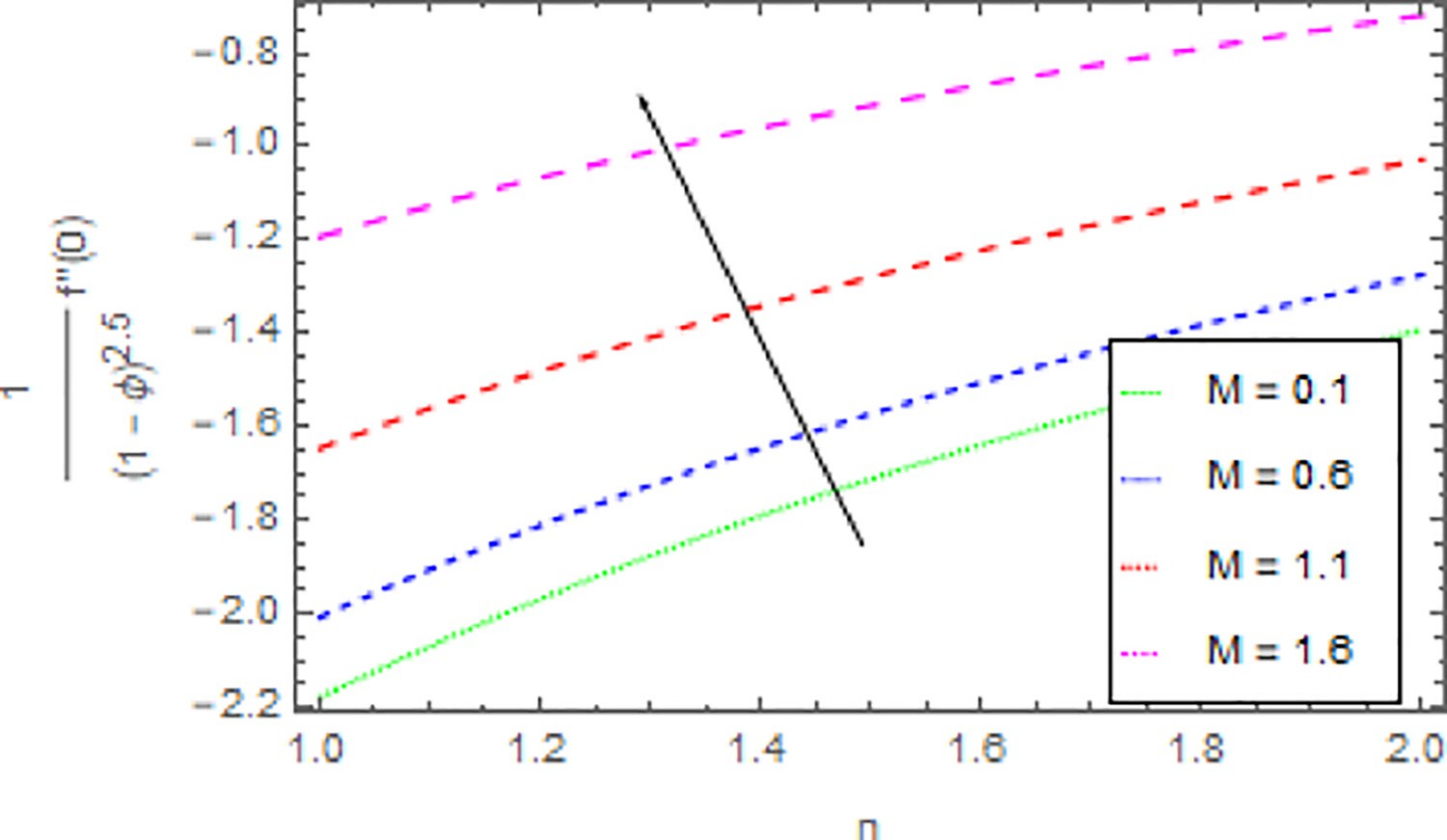
Deviation in skin friction coefficient of velocity (*x*−direction) against *M* and *n* when Pr = 1000, *m* = 0.10, *δ* = 0.40, *β* = 1.00, *Nr* = 0.20 and *ϕ* = 0.01 to *ϕ* = 0.04.

**Fig 17:**
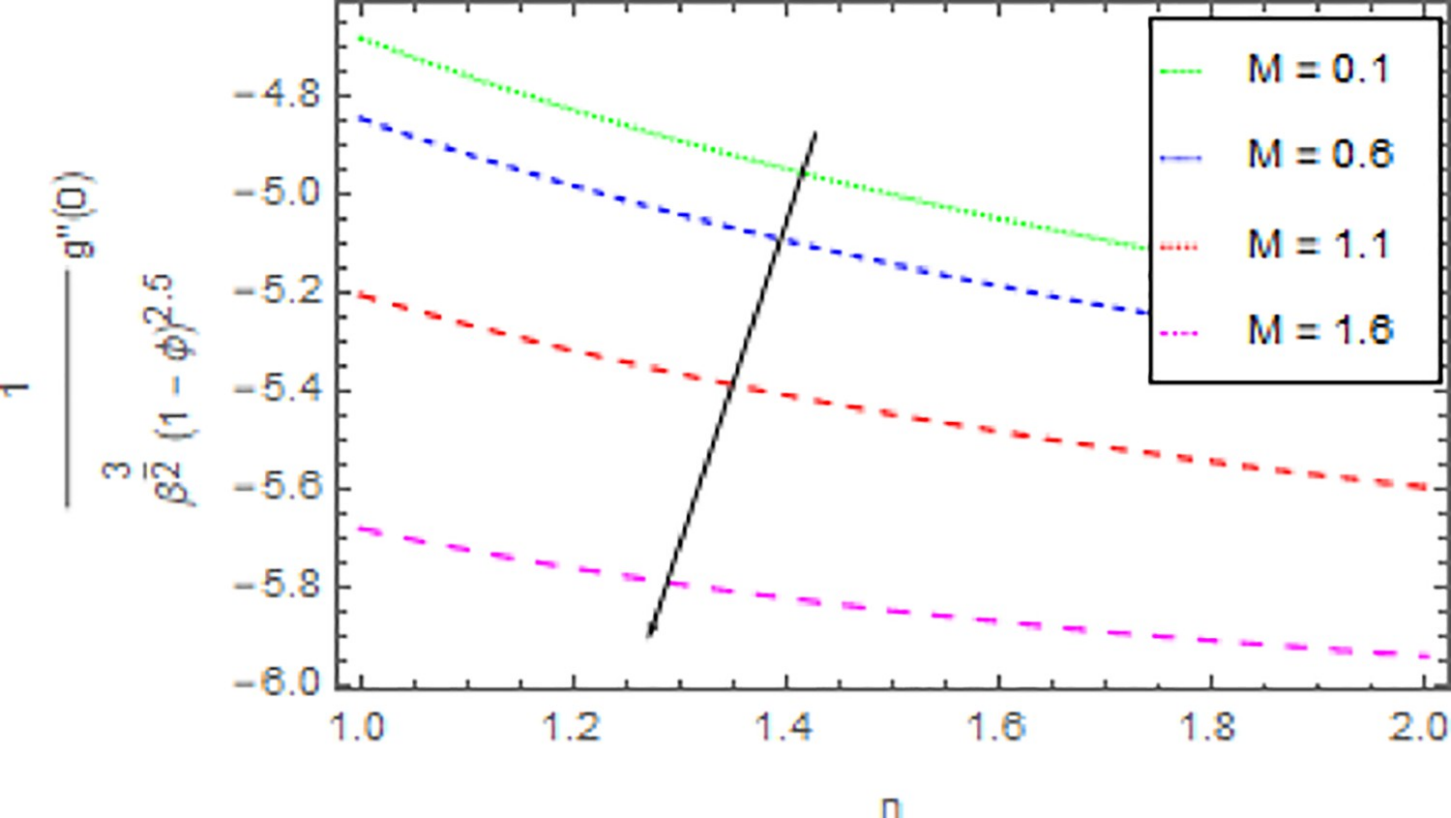
Deviation in skin friction coefficient of velocity (*y*−direction) against *M* and *n* when Pr = 1000, *m* = 0.10, *δ* = 0.40, *β* = 1.00, *Nr* = 0.20 and *ϕ* = 0.01 to *ϕ* = 0.04.

**Fig 18:**
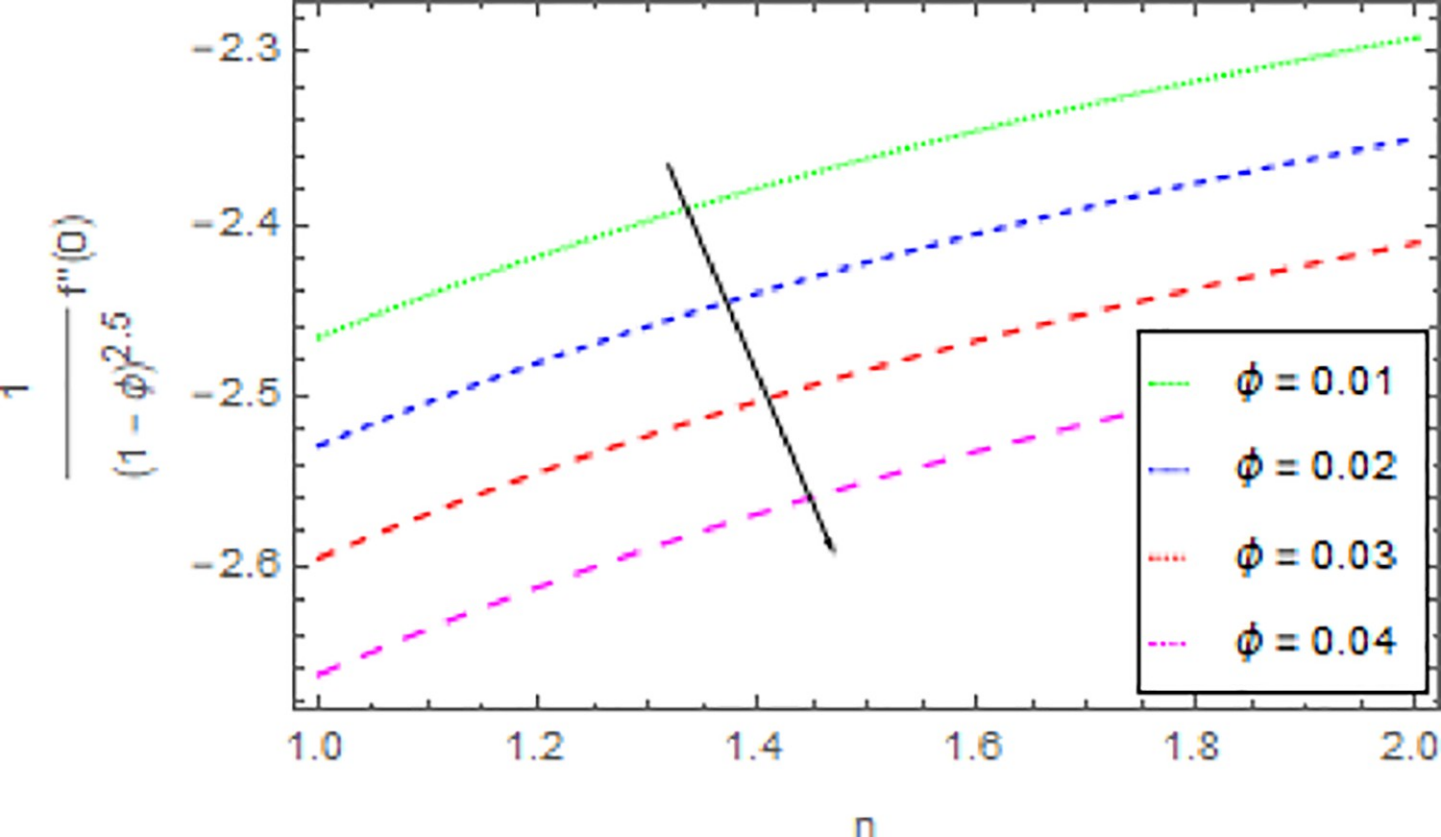
Deviation in skin friction coefficient of velocity (*x*−direction) against *ϕ* and *n* when Pr = 1000, *m* = 0.10, *M* = 2.50, *δ* = 0.40, *β* = 1.00 and *Nr* = 0.20.

**Fig 19:**
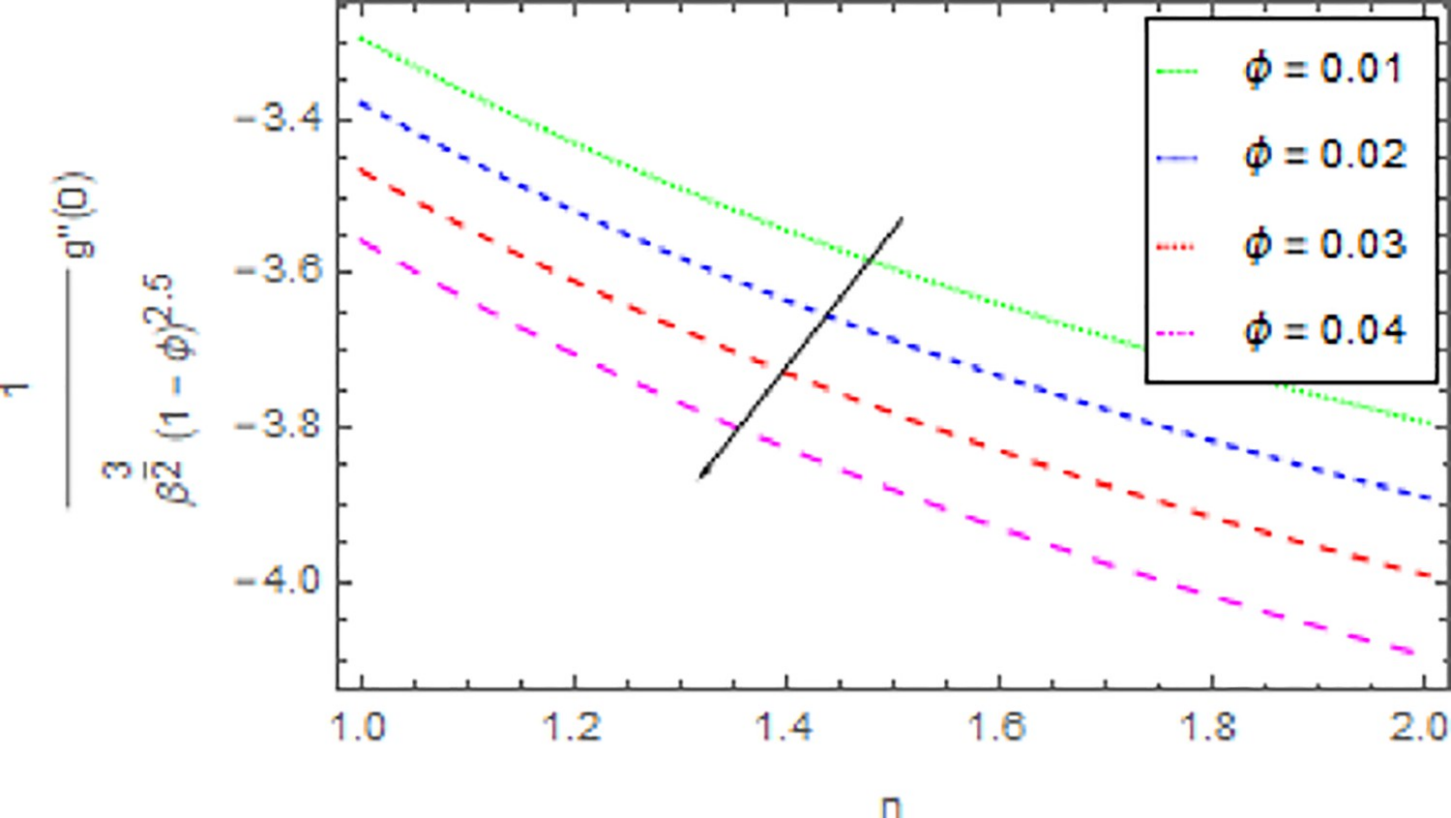
Deviation in skin friction coefficient of velocity (*y*−direction) against *ϕ* and *n* when Pr = 1000, *m* = 0.10, *M* = 2.50, *δ* = 0.40, *β* = 1.00 and *Nr* = 0.20.

The variation in skin friction coefficient profile *Cf*_*x*_ and *Cf*_*y*_ of the nanofluid through different flow parameters are enlightened in Figs 16–19. Fig 16 predicts the graph of *Cf*_*x*_ in *x*−direction via shape parameter *n* for several estimation of *M*. It is detected that the increment in *n* and *M* led to enhance the *Cf*_*x*_ in *x*−direction. For varied values of magnetic parameter *M* the *Cf*_*y*_ in *y*−direction as a function of shape parameter *n* is revealed in Fig 17. From this observation, it is clear that when *n* and *M* are rises then the *Cf*_*y*_ in *y*−direction is reduced. Fig 18 present the consequence of the shape parameter *n* on the *Cf*_*x*_ in *x*−direction via shape parameter *n* for higher estimations of the *ϕ*. The decline in *Cf*_*x*_ is examined for larger values of *n* and *ϕ*. Another experimental study in Fig 19 is made on *Cf*_*y*_ in *y*−direction via shape parameter *n* for distinct estimation of *ϕ*. It is sensed that with the enhancing of *ϕ* and *n*, the *Cf*_*y*_ in *y*−direction iss decreased.

## 6. Conclusion

In this article, the consequence of Hall current and Joule heating effects on the three-dimensional magnetohydrodynamics flow of nanoliquid with Cattaneo-Christove heat flux in a stretching surface has been deliberated. Additionally, the impact of thermal radiation is studied. This problem is related to the study of vacuum pump oil in the pumps of different machines therefore in this investigation vacuum pump oil (VPO) is taken as a base fluid and *Fe*_3_*O*_4_ is the nanoparticles suspended in an VPO. The basic equations of the modeled problem are modified with the assistance of the HAM scheme. The influence of distinct flow parameters over the velocities, temperature and skin friction coefficient profiles have been offered graphically. Numerical computation for *Cf*_*x*_, *Cf*_*y*_ and *Nu*_*x*_ are offered in a tabular form. The main findings of the current scrutiny are given below

- The amplification in skin friction coefficients *Cf*_*x*_ is observed for *n*, *M*, *ϕ* and *β* but *m* decreased the *Cf*_*x*_.
- Higher values of *ϕ* and *β* lead to enhance the *Cf*_*y*_ but the opposite trend is noted on skin friction coefficients *Cf*_*y*_ against *m*, *n* and *M*.
- It is distinguished that the *Nu*_*x*_ is improved with the augmentation of *m*, *M*, *ϕ* and *β*.
- Nusselt number *Nu*_*x*_ falls due to the rise of *n*, *Nr* and *δ*.
- Both the velocities in *x*−direction and *y*−direction of the nanofluid are rising for nanoparticles volume fraction *ϕ*.
- It is perceived that the nanoliquid velocity in *x*−direction is enhanced through the enlargement of *M*.
- The nanoliquid velocity in *x*−direction *f*’ against larger values of *n* and *m* is decayed.
- The velocity *g*’ in *y*−direction of the nanofluid is an expanding function of *M*, *n* and *m*.
- The expansion in *n* and *Nr* boosted the nanofluid temperature but the opposite reaction is notable on the nanofluid temperature against *M*, Pr and *δ*.
- Increment in *ϕ* enhanced the nanoliquid temperature.
